# Supplement to the First General Index

**Published:** 1820

**Authors:** 


### SUPPLEMENT

TO THE

### FIRST GENERAL INDEX.

### A.

ABDOMEN, account of a complete transposition of the viscera of the, xxxvii. 157, 346; case of the escape of the contents of the stomach into the cavity of the, xxxix. 154; observations on morbid condition of the viscera of the, as a cause of insanity, 452; account of an unusual distribution of the viscera of the, xl. 339; observations on the causes of pulsation in the, 529.

ABERCROMBIE, Dr. observations on certain dropsical affections that are alleviated by blood-letting, xxxix. 411; observations on inflammation of the brain, xl. 315.

ABSORPTION, proof of that of mercury by the skin, xxxv. 74; observations on, xxxvii. 60, 517; observations respecting that of some species of morbific poison, 7; a new hypothesis of the causes of, xl. 2.

ABSTINENCE, remarkable case of, xxxviii. 341.

ACAREI, some account of their natural history, xxxv. 461.

ACCUM, Mr. account of his Chemical Amusement, xxxviii. 422; chemical analysis of the Moira saline water, 428.

ACHARIUS, Dr. description of two new genera of lichens, xxxv. 244.

ACIDS, observations on the use of diluted sulphuric as a lotion in typhous fever, xxxv. 73; on the efficacy of muriatic, in the diarrhœa of children, ibid.; account of Sir H. Davy's discoveries respecting the muriatic, 111; observations on the use of citric acid in sea-scurvy, 257; observations on the efficacy of that of sugar in the cure of scurvy, 338; observations on the poisonous qualities of the oxalic, xxxvi. 517; observations on their effects on the animal fibre, xxxvii. 68; observations on the use of the mineral acids in the ment of syphilis, xxxviii. 429; observations on the use of nitro-muriatic acid bath in the cure of several diseases, 466; hydrioidic, experiments on, xl. 100; oxalic, experiments on, ibid.; experiments on citric, 338; pyroligneous, observations on, 354; prussic, on the mode of preparing it, 449; observations on the use of carbonic acid gas in typhus, 22.

ACUPUNCTURATION, observations on its use in some diseases, xxxviii. 436.

ADAMS, Dr. Joseph, observations on the practice of midwifery, xxxv. 84; account of the acareus insect, 466; on cancer of the uterus, xxxvii. 294; on epilepsy, xxxviii. 39; extracts from his life of John Hunter, 107; account of a case in which an ounce of sulphuric acid had been swallowed, 129; observations on the fevers of London, xxxix. 39; on muscæ volitantes, 103; observations respecting the history of vaccination, 265.

———, Sir William, observations on the means of remedying the conical shaped cornea, xxxvii. 211; on extraction of the cataract, xxxix. 126.

ADDER, account of one found in a block of coal, xxxvii. 431; effects of the bite of one on a man, xxxix. 161.

ADHESION, some remarks on the doctrines of, xxxvi. 420.—*See* WOUNDS.

AGARIC, an account of the deleterious effects of the A. *campanulatus*, xxxvi. 451.

AIR, observations respecting the influence of the, on the character of diseases, xxxv. 417; on the influence of peculiar states of the, in producing tetanus in the Caribbee islands, 475; observations on the means of purifying the, xxxvii. 167; account of some experiments on that of the fever-hospitals at Cork, xxxix. 274.

ALBERS, Dr. account of his treatise on the croup, xxxvi. 306; observations on a change of colour of the skin produced by the internal use of the nitrate of silver, xxxvii. 152.

ALCOHOL, account of a mode of obtaining it from potatoes, xxxviii. 432.

ALCYONIUM, description of the, xxxvi. 212.

ALDIS, Mr. observations on cancer, xxxvii. 271.

ALISMA PLANTAGO, account of it as a preventive of hydrophobia, xxxviii. 455; xl. 76, 453.

ALKALIES, observations on their efficacy in dyspepsia, xxxvii. 271; account of their agency on the animal fibre, 68; on their use in calculous disorders, xxxix. 320; on the influence of hydrogen on, xl. 105.

ALLAN, Mr. John, observations on the use of blood-letting in fever, xxxvi. 296.

ALLINHOEFER, observations on the nature and treatment of syphilis, xxxvii. 36.

ALUM, account of its use in tympanites, xxxv. 73.

AMAUROSIS, observations on the benefits of insulation in, xxxv. 157; account of a case of, arising from organic disease of the brain, 302; some observations on the origin of, xxxvii. 392. —*See* Eyes.

AMBLYOPIA, case of, arising from a wound, xxxvii. 280.—*See* Eyes, *and* Vision.

AMERICA, account of the origin of the yellow fever of, xxxvii. 39; xxxix. 149; account of the epidemics of, xxxviii. 165.

AMPHIBIA, some observations on the urinary secretions in some species of, xxxix. 514.

AMPUTATION, observations on Chopart's mode of partial amputation of the foot, xxxv. 74; xxxvi. 448; at the shoulder-joint, case of, xxxvi. 108; at the instep, case of, xxxvii. 359; on the use of silk ligatures after, 521; remarks on the proper period for performing it in cases of gun shot wounds, xxxviii. 62; at the shoulder-joint, case of, 359; at the foot, case of, xxxix. 523; remarks on the period proper for it in cases of gun-shot wounds, xl. 159; observations on the proper period for it after gun-shot wounds, 221; general and particular remarks on, 221.

ANATOMY, account of that of the parts concerned in congenital hernia, and of the descent of the testicle, xxxv. 109; account of that of the centres of the ganglionic system of nerves, 139; notices of an elementary treatise on, 508; observations on the formation of the bones, xxxvi. 157; observations on a new liquor for preserving preparations in, xxxvii. 180; remarks on the ordinary division of, 109; a review of the system of, of Dr. Wistar, xxxviii. 414; observations on the structure of the retina, xl. 178; remarks on the axis of the pelvis, 223.

ANATOMY, *comparative*, account of the structure of the feet of some lacertæ, xxxv. 333.

ANCHYLOSIS, of the spine, observations on, xxxvii. 158; of the knee-joint, xl. 222.

ANEURISM, case of spurious inguinal, in which the operation of tying the iliac artery was resorted to, termination fatal, xxxv. 368; case of, in the orbit of the eye, in which the trunk of the carotids was tied with success, 411; case of axillary, in which the subclavian artery was tied with success, 412; case of femoral, xxxvii. 154; account of an improved operation for, 377; case of aortal, 420; on the method of operating for external, 500; case of inguinal, cured by tying the iliac artery, 509; cause of spontaneous cure of an inguinal, xxxviii. 60; history of a very remarkable case of, of the heart, 186; case of gluteal, for which the internal iliac artery was tied, 267; observations on aneurisms of the heart, xl. 443, 444; case of, of the femoral vein, from gunshot, which terminated fatally from ligature of the femoral artery, 215; case of, of the iliac artery, for which a ligature was made of the aorta, 511; cases of, of the brachial and carotid arteries, treated successfully by the ligature, 531.

ANGINA PECTORIS, case of, relieved by inhalation of oxygen gas, xl. 303.

ANGUSTURA, account of the poisonous effects of the spurious species, xxxvi. 162.

ANIMALS, observations on the functions of, and their division into those of organic and relative life, xxxv. 42, 119; observations respecting the mode of conducting physiological experiments on, 460; observations on experiments on, xxxvi. 196; experiments and observations on the tenacity of the fibres of, xxxvii. 63; observations on experiments on, 110; observations on the influence of the atmosphere on, 167; remarks on Bichat's division of the frame of, into the organs subservient to relative, and to organic, life, 59; some remarks on the functions of, in general, xxxviii. 78; observations on the medical measures instinctively resorted to by brute animals, xl. 486.

ANKLE, account of the removal of some of the bones of the, xxxvii. 522; for amputation at the, *see* AMPUTATION.

ANTIMONY, observations on the antimonial powder of the London Pharmacopœia, xxxv. 345; observations on a new mode of preparing the tartrite of, 513; observations on the use of the tartrite of, in inflammation of the testicles, xxxvi. 286; observations on the efficacy of the oxide of, in removing the apoplectic diathesis, xxxix. 498; observations on the medicinal qualities of the tartrite of, 73, 410, 413; xl. 410.

AORTA, case of aneurism of the, xxxvii. 420; case of obliteration of the, 527; account of the obliteration of the, in dogs, 99.—*See* ARTERIES, *and* ANEURISM.

APOPLEXY, case in which apoplectic symptoms accompanied the passing of a large gall-stone through the biliary duct, xxxix. 441; observations on the use of James's Powder in obviating the diathesis to, 498.

APOTHECARIES, remarks on the laws respecting, xxxv. 19; report of the general committee of, in the year 1816, 427; observations respecting the qualification of, xxxvi. 428; observations on the Act relative to, xxxvii. 490; address to the society of, 78; circumstances respecting the Acts relative to, 489, 490; xxxviii. 249; report of the general committee of, in 1817, 252; in 1818, xxxix. 256; in 1818, xl. 167.

ARABS, account of the customs of the, relating to parturition, xxxvii. 257.

ARETÆUS, his account of elephantiasis, xxxviii. 297.

ARM, case of a disease of the, similar to the Barbadoes leg, xxxvii. 239.

ARMSTRONG, Dr. John, account of his work on typhus, xxxvii. 305; account of his work on scarlatina and chronic diseases, xxxix. 499.

ARMIGER, account of his Rudiments of Anatomy, xxxv. 508.

ARMY, hints to the medical officers of the, xxxviii. 441; observations relative to the practice of surgery in the, xl. .

ARSENIC, observations on the poisonous effects of, and on the mode of detecting it in the animal fluids, xxxv. 337, 338, 394; xxxvii. 430; xxxviii. 92, 240, 252, 448, 172; xxxix. 28, 58, 76, 174, 189, 343, 376; xl. 108, 281, 407.

ARTERIES, observations on the state of, in inflammation, xxxv. 13; experiments and observations on the connexion of the action of, with the influence of the nervous system, 120, 122; observations on the application of ligatures to, after operations, 190; observations on inflammation of the, and on coagula formed in, 234; on the state of the coronary arteries during the systole of the heart, 262; observations on the irritable state of the, during fever, 355; observations respecting the state of the, during spontaneous hæmorrhage, 450; experiments and observations on the cause of the pulsative motion of the, 478; observations on the tonic powers of the, 481; some observations respecting the generation of new ones, and on the doctrine of their containing blood, xxxvi. 94, 137; observations on a new mode of tying the, after various operations, 227, 235; two cases of wounds of, terminating without the formation of aneurism, 332; case of obliteration of the aorta, xxxvii. 527; observations on the reproduction of obliterated, 99; case of ligature of the carotid, 152; case of wound of the peroneal, 509; case of extraordinary disease of the humeral, 508; observations on their eruption after death, 55; observations on the tonic power of the, 56; observations on the functions of the, 99; account of the effects of different methods of obliterating the, 500; observations on the functions of the, 517; delineation of the carotid of a ram, 100; observations on the motion of, xxxviii. 11, 12, 13, 14; remarks on the consequences of the ligature on, 68, 69; case of ligature of the internal iliac, 267; observations on morbid excess of distension of the, 373; remarks on Mr. Cooper's case of ligature of the aorta, xxxix. 180; remarks on inflammation of the, 7; on ligatures of the, 427; cases of obliteration of the aorta, xl. 515; observations on injuries of the, from gun-shot, 214; case of, causing varicose aneurism, which terminated fatally from ligature of the artery, 215; case of ligature of the aorta, 511; cases of ligature of the brachial and carotid, 531.

ASHBURNER, Dr. some observations on the saliva of rabid animals, xxxvi. 337.

ASTHENIA, some observations on the phenomena resulting from, xxxvii. 344.

ASTHMA, account of the appearances on dissection in a case of humoral, xxxvi. 332; observations respecting its most frequent origin, xxxvii. 394.

ASTRINGENTS, general botanical account of the species of, xxxvi. 343.

ATKINSON, Mr. remarks on the propriety of obstetric practice, by men, xxxv. 3; xxxvi. 96, 452; account of the application of a ligature to the internal iliac artery, xxxviii. 267; observations on uterine hæmorrhage, 462.

ATMOSPHERE, observations on the exclusion of the, from the surface of the body, by the use of oiled silk, xxxv. 169; observations respecting its influence on the human system, xxxvii. 167; account of a general treatise on its influence on the human system, xxxix. 247; some remarks of Sydenham on the constitution of the, 262.

AUSTIN, Mr. case of periodical amaurosis, xl. 267.

AUTENREITH, Prof. observations on the causes of deafness, xxxvi. 486.

AUTOMATIC MOTION, observations on, xxxvii. 135.

AZOTE, observations on its influence on the qualities of the urine, xxxvii. 343.

B.

BACON, of Verulam, his observations on the influence of language, xxxv. 142.

BACOT, Mr. sketch of the medical history of the first battalion of the foot-guards in 1814, 1815, xxxvii. 325.

BAGNOLD, Captain, observations on the wind of a cannon-ball, xxxviii. 265, 373.

BAILEY, Mr. observations on the use of belladonna in neuralgia, xxxix. 141.

BAINES, Mr. observations on the means of remedying damaged corn, xxxvii. 80.

BALFOUR, Dr. Wm. review of his work on rheumatism, xxxvi. 318, 445, 447; on re-union by the first intention, 420; on the cure of gout, ibid.; remarks on the review of his work on Rheumatism, xxxvii. 199; on re-union of divided parts, 472; on the cure of whitlow by compression, xxxix. 187; review of his work on the sedative and febrifuge powers of emetic tartar, xl. 410; on the efficacy of nitrate of silver in the cure of several diseases, 453.

BARBADOES LEG, description of the, xxxv. 393.

BARLEY, analysis of, before and after germination, xxxix. 258.

BARLOW, Mr. account of an improved sound for the bladder, xxxv 81.

BARNARD, Sir Thomas, observations on the salt duties, xxxix. 289.

BARON, Dr. remarkable case of rupture of the brain from hydrocephalus, xxxviii. 404.

BARTLETT, Mr. remarkable case of extra-uterine gestation, xxxv. 278.

BARTON, Dr. observations on the impurities of water, xxxvii. 397.

BATEMAN, Dr. on the fever of London, xxxix. 34.

BATHS, observations on the use of vapour, and a new apparatus for, xxxv. 172; account of the warm, used by the aborigines of America, 250; observations on the good effects of warm, in the first stage of fever, xxxvi. 156; observations on the use of medicated, in cutaneous diseases, xxxvii. 527; observations on the use of nitro-muriatic acid baths in several diseases, xxxviii. 466.

BAYLE, M. some account of his life and character, xxxv. 513.

BECLARD, M. account of a case of the general transposition of the thoracic and abdominal viscera, xxxvii. 157, 346; account of a female dwarf, xxxix. 258.

BEDINGFIELD, Mr. review of his Medical Compendium, xxxvi. 402; xxxvii. 218.

BEECH, Mr. account of the gas produced from coal-tar, xxxvii. 329.

BEER, observations respecting the fermentation of, xxxix. 302.

BELL, Mr. Charles, observations on the effects of ligature on arteries, xxxviii. 13; review of his Surgical Observations, 476; remarks on his opinions respecting the cure of syphilis, 235; review of his Surgical Observations, xl. 48, 139, 222.

———, Mr. John, review of his Treatise on Surgery, xxxvii. 351.

BELLADONNA, observations on its use in neuralgia, xxxix. 176; observations on its medicinal qualities, xl. 139.

BENT, Dr. observations on the varioloid disease prevalent in Derby, xl. 458.

BERLIOZ, Dr. observations on the efficacy of acupuncturation and other modes of sanguineous evacuation, in various chronic diseases, xxxviii. 436.

BERNE, Dr. observations on the mode of applying leeches, xxxv. 375.

BERTHOLLET, M. history of a case of demonomania, xxxv. 74.

BICHAT, M. some account of his researches on life and death, xxxv. 41, 125; remarks on his opinions respecting inflammation, xxxvii. 59; remarks on some of his physiological opinions, xxxviii. 7, 8, 9.

BILIARY DISORDERS, some remarks on the causes and treatment of, xxxvii. 145.

BILIARY DUCTS, case of remarkable enlargement of the, xxxix. 499.

BIOGRAPHY, of Dr. Denman, xxxv. 62; of Dr. Lettsom, 204; of M. Bayle, 513; of Dr. John Squire, xxxvi. 257, 347; of Dr. Ingenhouz, xxxviii. 377; of Dr. William Saunders, 388; of Dr. C. Combe, 392; of Dr. Wells, 395; of Dr. Werner, 397; account of a work called a Picture of the present State of the Royal College of Physicians in London, 403; of Dr. Woodville, xl. 34; of Dr. Rush, 39.

BIRNIE, Mr. some observations relative to diseases of seamen, xxxviii. 144.

BISSEL, Dr. account of a change of the colour of the skin from black to white in an Indian, xl. 391.

BITTERS, observations on their agency on the animal fibre, xxxvii. 67.

BITUMEN, mode of preparing the oil of, xxxvii. 339.

BLACK, Dr. case of gouty affection, xl. 300; account of the appearances on dissection of two habitual drunkards, 299.

BLACKADDER, Mr. review of his work on Phagedœna Gangrænosa, xl. 226.

BLADDER, observations on the mode of sounding it, xxxv. 81; cases of various species of disease of the, 218; case of wound of the, xxxviii. 346; observations on diseases of the, connected with disorders of the alimentary canal, xl. 145.

BLAGDEN, Mr. account of a case of fatal hæmorrhage from the extraction of a tooth, xxxix. 76.

BLAKE, Dr. observations on the influence of language on ideas, and on the present state of medicine, xxxviii. 274.

BLEEDING, case of copious in a youth, in a case of fever, employed with success, xxxv. 79; case of copious in a child, for the cure of croup, employed with success, ibid.; observations on the use of, in peripneumony, 105; observations relative to the use of, in diseases of the brain, 305, 307, 310; observations on the use of, in children, in inflammation of the membranes of the brain, 340; observations on, from an early English work on surgery, 381; observations respecting the use of copious, in the treatment of tetanus, 412; on the use of, in gout, 414; on the extensive use of, in inflammation of the larynx, 413; on its use in a fever prevalent in the British fleet, 499; general observations on the indications of its use in fever, 502, 505; extensive use of, in diabetes, without being productive of benefit, xxxvi. 26; its successful use in a case of supposed rabies in a dog, 71; observations on, for rabies in dogs, 72; two cases of its successful use in hydrophobia, 74; observations on its beneficial use in diabetes, 115; remarks on the efficacy of free, in fever, 156; observations respecting the necessity of copious, in some inflammatory diseases, 234; observations on its utility in fever, 246; observations on the use of copious, in violent contraction of the uterus during pregnancy, 331; observations on the advantages of local, in some cases, 364; observations on the use of free, in the treatment of fever, 368, 369, 370; observations on its use in gout, 505; observations on, xxxvii. 12; observations on its use after severe injuries, 97; on its use in diabetes, 104; on its use in hooping-cough, 223; observations on its utility in the cure of rheumatism, 314; observations on the use of small and repeated, in chronic hepatitis, xxxviii. 33; observations respecting its use in some cases of dropsy, xxxix. 411; observations on the efficacy of bleeding from the region of the arm in some diseases, 406; observations on the efficacy and propriety of copious blood-letting in fever, 55; caution respecting its use in fever, 485; observations on the excessive use of, in mania, xl. 368.

BLEGBOROUGH, Mr. H. observations on the treatment of croup, xxxviii. 126.

BLISS, Dr. observations on cynanche laryngea, xl. 336.

BLISTERS, observations on their use in general, xxxviii. 102.

BLOOD, observations on the respective influence of red and black blood on different parts of the system, xxxv. 127, 130, 131; on the change of colour of, in the lungs, 133; observations on the influence of galvanism on the, 332; observations on the buffy coat of the, xxxvi. 254; observations on the Harveyan doctrine of the circulation of the, xxxvii. 99; observations on the momentum of the, in the arteries, 51; account of the state of the, in diabetes, 105; on the causes and consequences of excessive determination of the, 135; on defective determination of the, 139; on the dangerous effects of extravasated, 256; on the evolution of heat during the coagulation of the, 387; remarks on Hunter's doctrine of the vitality of the, xxxix. 254; observations on the colouring matter of the, xl. 118.

BLOW-PIPE, account of some improvements in the construction of the, xxxv. 510; xxxix. 520; xl. 91.

BOGGIE, Mr. case of gun-shot wound and fracture of the tibia, xxxvii. 500.

BONES, observations on the formation of the, xxxvi. 173; remarks on some carious affections of the, xxxvii. 158, 468; observations on their structure, 325; history of a peculiar affection of the humerus, xxxviii. 355; xl. 364; observations on morbid structure of the, xxxix. 147; observations on injuries of the, from gun-shot wounds, xl. 162, 165; observations on exostoses, 516.

BOSTOCK, Dr. observations on diabetic urine, xxxvii. 187.

BOTANY, reference to a *Flora* of St. Helena, xxxv. 337; observations respecting Mr. Salisbury's mode of teaching, 430, 516; xxxvi. 83, 170; review of a practical treatise on, xxxvii. 248.

BOUGON, Mr. case of strangulation of the intestines, xxxviii. 169.

BRAIN, observations on the probable consequences of concussions of the, and the means of obviating them, xxxv. 10; observations on determination of blood to the, 11; observations on its functions, 46; experiments and observations on its influence on the sanguiferous system, 120; on the consequences of the cessation of the action of the heart on the functions of the, 127, 138; observations on inflammation of, in children, 241; on the consequences of metastasis of diseased action to the, 268; cases, illustrative of the pathology of the, 295; observations on the importance of its influence to the action of the heart, 317; observations on the peculiar frequency of inflammation of the membranes of the, in children, 339; case of what has been termed hernia of the, xxxvi. 449; observations on the effects of extravasation on the, xxxvii. 77; case of chronic inflammation of the, 174; observations on the consequences of excessive determination of blood to the, 135; on defective determination of blood to the, 139; observations on the formation and structure of the, 290, 349; on Gall and Spurzheim's mode of demonstration of the, 427; observations on the structure of the, 428; some remarks on the influence of intestinal disorders on the, xxxviii. 26, 127; observations on the use of cold applications to the head, in inflammation of the, 103; case of rupture of the, from hydrocephalus, 464; observations on inflammation of the, as a cause of typhous fever, 491; remarks on sympathetic affection of the, xxxix. 4, 498; various observations respecting its functions, 53, 573; observations on concussion of the, 457; observations on its state in fever, 50, 53, 63; observations on the varieties of inflammation of the, xl. 315; observations on solution of continuity of the, as a cause of paralysis, 351; observations on its absence in the fœtus, and on the causes of this phenomenon, 431; some observations respecting its influence on the functions, 439; case of severe disease of the, originating from ictus solis, 447; case of inflammation of the, 474; observations on the varieties of inflammation of the, 315.

BRANDE, Mr. observations on meteoric stones, xl. 136.

BRANDRISH, Mr. account of a case in which a very large quantity of opium was taken without any sensible effects, xxxix. 449.

BREAD, some observations relative to the making of, especially from damaged corn, xxxvii. 80, 160.

BREEN, Dr. observations on the turning of the fœtus in the uterus, xxxix. 159.

BRES, Mr. observations on animal heat, xxxvii. 283.

BREWSTER, Rev. J. account of the sleeping woman of Dundonald, xxxv. 509.

BRINE, Dr. observations on the fevers of London, xxxix. 168.

BRODIE, Mr. observations on the treatment of varicose veins of the legs, xxxvii. 155.

BRONCHITIS, observations on, xxxvii. 221.

BRONCHOTOMY, case of, xxxv. 419.

BROOKE, Dr. of Dublin, history of a case of hydrocephalus, xl. 301; histories of three cases of melæna, 304.

———, Mr. account of his improvement in the blow-pipe, xxxv. 510.

BROOKES, Mr. J. account of the life of Mr. Royston, xxxvii. 114.

BROWN, Dr. John, remarks on some parts of his doctrine, xxxviii. 6.

———, Dr. William, on the use of blood-letting from the region of the anus in some diseases, xxxix. 406.

BROWNE, Mr. account of a case of recovery of a person who had swallowed a large quantity of sulphuric acid, xl. 25

BRUGNANS, Professor, conclusion of his memoir on hospital gangrene, xxxv. 20.

BUCK, Drs. W. and S. history of some cases, showing a family predisposition to hæmorrhage, xl. 429.

BURROWS, Dr. account of the statement of the circumstances connected with the Apothecaries' Act, xxxvii. 488; on the medical cure of the poor, xxxix. 332.

BUSH, Mr. observations respecting vaccination, xxxviii. 107.

BUXTON, Dr. remarks on climate and on consumption, xxxvii. 360; xxxviii. 97.

C.

CADET, M. an account of the Materia Medica of the Galibis and Guaripons, natives of Guinea, xxxvi. 341; on the honey of Mount Hymettus, xxxvii. 85.

CADIZ. some topographical observations relative to, xxxvii. 237.

CAIRO, account of the plague at, xxxviii. 477.

CALABRIAN ASH, some observations respecting the juice of the, xxxix. 327.

CALCULI, various observations on the causes and treatment of urinary, xxxvi. 69; xxxvii. 313, 448; xxxviii. 36; xxxix. 313, 448; cases where the stone was extricated through the urethra, xxxix. 417; xl. 545.

CALLANAN, Dr. review of his treatise on fever, xl. 491.

CALOMEL, observations respecting its use in some disorders of children, xxxv. 340; remarks on its use in fever, 407; two cases of hydrophobia, in which calomel was employed with apparent benefit, xxxvi. 74; observations on its use in dysentery, xxxvii. 314; account of a new mode of preparing it, xl. 106.

CALVERT, Dr. account of the plague at Malta, xxxv. 319.

CANADA, account of the cutaneous disease epidemic in, xxxix. 209.

CALDWELL, Dr. account of his commentaries on Miller's Practice of Medicine, xxxviii. 155.

CANCER, observations on that of the uterus, and on extirpation of that organ by the knife, xxxv. 426; observations on, affecting the uterus, xxxvi. 250; observations on the sympathy between that of the uterus, and that of the breast, 293; further observations on that of the uterus, and on excision of the organ for, 295; general observations on its nature and origin, 459; on the use of pressure in the treatment of, 461; observations on the treatment of by pressure, xxxvii. 354; report of the committee at the Westminster Hospital respecting the use of pressure as a remedy for, xxxviii. 478; case of, cured by arsenical application, xxxix. 518; observations on that of the prepuce, 306; case of, terminating in locked-jaw, xl. 336.

CAPILLARY TUBES, some observations on the rise of fluids in, xxxvii. 426.

CARPENTER, Mr. remarkable case of strangulated hernia, xxxvi. 453.

CARPUE, Mr. remarks on his success in performing the talliacotian operation, xxxix. 16.

CARSON, Dr. some notice of his work on the circulation of the blood, xxxv. 55; xxxvii. 155; xxxviii. 10.

CAUTERY, account of the use of, by the older English surgeons, xxxv. 387.

CATARACT, observations on the efficacy of galvanism in removing, xxxvi. 510; observations on the removal of that connected with the conical-shaped cornea, xxxvii. 211; account of a new method of operating for the, 216, 273; remarks on the operations for the, 455; account of Prof. Scarpa's improvements in the operations for, 496; various observations respecting the, xxxvii. 211; 216, 273, 455, 496, xxxviii. 338, xxxix. 127, 515.

CELSUS, his description of the methodic sect of Physicians, xxxvii. 235.

CHAMBERLAIN, Mr. case of ligature of the subclavian artery, xxxv. 412.

CHANCRE, remarks on the character of, xxxix. 235.—*See* Venereal Diseases.

CHANNING, Dr. observations on spontaneous hæmorrhage, xxxv. 450.

CHARNOCK, Mr. account of the composition and medical qualities of the Cartmeal waters, xxxvii. 267.

CHAUMETON, M. observations on forensic chemistry, xxxvi. 429; xxxvii. 165; extracts from his Medical Bibliography, 168, 208.

CHEMISTRY, review of Mr. Crowe's Chemical Table, xxxv. 59; account of the discovery of chlorine and iodine, 111; analysis of some aerolites, 423; account of an improvement in the blowpipe, 510; remarks on its application to physiology, xxxvi. 125; observations on the nature of chyle and chyme, 237; observations on the crystallization of pure lime, 255; observations respecting its application to physiology, 340; analysis of the Moira saline water, 428; observations on, relating to medical jurisprudence, 429; analysis of the urine of gouty patients, 503; observations on the state of the blood in diabetes, xxxvii. 105; observations on, relating to medical jurisprudence, 165; on its connexion with physiology, 209; observations on its application, especially to medicine, xxxviii. 1, 2, 3, 4; observations on the tests for arsenic, 92, 172, 242, 252, 446; various observations on, as connected with the labours of Baron Guyton de Morveau, 382; directions for various operations and experiments in, 422; analysis of the air of a fever-hospital, xxxix. 274; account of an improved method of using the blow-pipe, 520; observations on animal and vegetable, 16; account of the recent discoveries in, xl. 89.

CHEVALIER, Mr. account of a case of croup in which bronchotomy was performed, xxxv. 417.

CHEYNE, Dr. (author of the *English Malady*), his account of the man who could stop the action of his heart, xxxvi. 394.

CHEYNE, Dr. John, medical report of the Hardwicke Fever Hospital, in Dublin, for the year 1816-17; xxxix. 483; case of melæna, with observations on the alternate excess of morbid action in the mucous and serous membranes, 495; a case of jaundice, unaccompanied with any discoverable disease of the liver, or turgescence, or obstruction of the biliary ducts, 496; on the virtues of James's Powder in the apoplectic diathesis, 498.

CHILDREN, observations on the use of muriatic acid in the cure of the diarrhœa of, xxxv. 73; observations relative to the use of bleeding in, in the cure of croup, 79; commentaries on some of the most important diseases of, 235; on the ages of the greatest mortality of, 236; on the advantages of hired wet-nurses for, 239; on the convulsive diseases of, 240; on the pleuritis of, 241; on the strabismus of, 241; on the paralysis of the limbs of, 242; remarks on the most common diseases of, 329; on the most appropriate purgation for, ib.; on scrofula in, ib.; on their peculiar liability to inflammation of the membranes of the brain, 339; account of the tetanus of, in the Caribbeans, 475; statistical observations on the weight and size of new-born, xxxvi. 220; observations on the most perilous ages of, and the circumstances rendering them so, 221, 222; remarks on the mode of feeding them in the want of a nurse, 222; observations on inflammation of the trachea in, 306; account of a fatal affection of the pudendum of female, 482; general observations on the diseases of, xxxvii. 351; observations on a species of ophthalmy especially affecting them, xxxvii. 185; observations on the trismus of new-born, especially on its connexion with disease of the umbilical cord, xxxix. 497.

CHIRURGICUS, observations on the preference of local bleeding to venesection, xxxvi. 364; reply to, xxxvii. 12.

CHISHOLM, Dr. on the statistical pathology of Bristol and of Clifton, xxxviii. 133; some of his observations on yellow fever, xxxvii. 42.

CHLORINE, some observations on its nature, xxxv. 111; new researches on the nature of, xv. 93, 100.

CHOPART, remarks on his mode of amputating at the instep, xxxv. 74.

CHOREA, observations on the efficacy of colchicum in the cure of, xxxvi. 181; further observations on, 292; observations on the treatment of, 511.

CHRESTIEN, Dr. observations on the medical properties of gold, xxxix. 515.

CHYLE, observations on its chemical composition, xxxvi. 237.

CINCHONA, observations on its utility in the cure of the plague, xxxv. 152; remarks on its efficacy in the cure of scrofula, 329; case of periodical amaurosis cured by, xl. 267; observations on its effects on the animal fibre, xxxvii. 67, 68; a mode of preparing an excellent tincture of it, 84; account of a new preparation of, xxxviii. 81.

CIRCULATION, observation on the causes which derange the ordinary balance of the, xxxv. 12; observations respecting a new theory of the, 55; experiments and observations on its relations to the functions of the nervous system, 119; observations on derangement of the, from diseases of the heart, 147; observations on the chief agents of the, 260; on the influence of the brain on the, 317; some objections to the Harveyan doctrine of the, xxxvi. 94; observations on the increased heat produced by inordinate celerity of the, xxxvii. 53; observations on the different states of the, and the consequences of those variations, 135; observations on the Harveyan doctrine of the, 99; some observations on it, especially relating to its causes, xxxviii. 10, 11; on the influence of the active dilatation of the heart on the, 257.

CLANNY, Dr. an account of his safety-lamp for coal-mines, xxxv. 243.

CLARK, Mr. case of inflamed testis, treated by very copious bleeding, xxxvi. 178.

CLARKE, Mr. notice of his work on the gripes of horses, xxxvii. 145.

———, Dr. report of the lying-in hospital in Dublin, xl. 401.

CLIMATES, observations on that of New-Orleans, xxxv. 398; on the influence of tropical on the liver, and in the production of dysentery, 402; observations relative to their influence on the system, especially in respect to the production of phthisis, xxxvii. 360, 456; remarks on the endemics of tropical, 233; observations on the influence of different, on consumption, xxxviii. 97; observations respecting that of the north-eastern part of Asia, xxxix. 253; remarks on the imputed deterioration of that of England, 522; some observations on the state of, tending to the production of yellow-fever, xl. 205.

CLINE, Mr. extract from his Hunterian oration, xxxv. 249.

CLOQUET, M. description of a singular case of dropsy of the periosteum, with a detachment of the epiphyses, in a hydrocephalic fœtus, xxxix. 197.

CLUBB-FEET, account of a new mode of remedying them, xxxix. 489.

CLUTTERBUCK, Dr. case of diseased action of the heart, effectually relieved by blood-letting and confinement to a horizontal posture, xxxviii. 130.

COBALT, account of the discovery of it, in meteoric iron, xxxvii. 343.

COGAN, Dr. some account of the life of, xxxix. 346.

COLCHICUM, accounts of its medicinal qualities in various diseases, xxxv. 510; xxxvi. 181, 292, 444; xxxvii. 17, 81; xxxviii. 76, 428; observations on its use in gout, xl. 396.

COLD, observations respecting its effects on the animal economy, xxxv. 400; some remarks on the cooling treatment of gout, 366; observations on the superior efficacy of cold applications in the treatment of burns, xxxvi. 239; observations on the use of cold affusion in fever, xxxvii. 311; observations on the medical use of, xxxviii. 102; observations on the use of cold applications to the head in typhous fever, 278; observations on the use of cold applications in gout and rheumatism, 283; case illustrative of the good effects of cold air, &c. in typhous fever, 433; observations on the use of cold water in fever, xxxix. 56, 485; observations on the use of cold application to burns, xl. 417; observations on its use in fever, 313.

COLDEFY, M. account of some new preparations of ipecacuanha, bark, and rhubarb, xxxvii. 83.

COLERIDGE, Mr. observations on the small-pox, xxxvi. 517.

———, Rev. Mr. history of a case of secondary small-pox, xxxvii. 27.

COLLECTANEA MEDICA, memoir on the state and composition of the atmosphere, considered as the cause of the hospital gangrene, xxxv. 20; report of the Committee on the state of madhouses, 28; observations on hernia congenita, 109; observations respecting chlorine and iodine, 111; biography of Dr. Lettsom, 204; two cases of elephantiasis, 210; cases illustrative of the pathology of the brain, 295; observations on leprosy, from a black-letter treatise on surgery, 311; on the use and manner of bleeding, from the same work, 381; history of a case of the disease termed the Barbadoes leg, 392; extract from Kirby's and Spencer's introduction to Entomology, 466; extract from the Abbé Raynal's history of the European settlements in the East and West Indies, and relating to tetanus and yellow fever, 475; history of a case of secondary small-pox, 475; parliamentary proceedings respecting the regulation of madhouses, xxxvi. 33; Dr. Suttaby's case of diabetes, 114; Dr. Jackson's reflections on the properties of life, 120; observations on the sea long worm of Borlase, by the Rev. Mr. Davies, 207; a tabular view of the external character of four classes of animals which Linnæus arranged under INSECTA, &c. by Dr. Leach, 209; observations on phthisis pulmonalis, by Sir James Macgregor, 214; description of a fossil alcyonium, by Mr. Mantell, 212; observations on cancer, 293; observations on Sir Everard Home's theory of the formation of fat, by Mr. Wadd, 303; on the clavus or ergot rye, and other plants, by Dr. Bigelow, 376; observations on disorders of the eyes, by Dr. J. C. Warren, 382; a case of water in the head, unattended by its usual symptoms, with observations, by Dr. Heberden, 388; observationes quædam de Hottentotis, præsertim de structura genitalium peculiari Hottentotarum auctore Dr. Somerville, 466; observations concerning the medical properties of the pyrola umbellata, and the arbutus uva ursi of Linnæus, by Prof. Smith Barton, 471; on the medical properties of the ergot rye, 474; observations on rabies, 477; observations on the nature of the syphilitic poison, xxxvii. 36; French report on yellow fever, 38; biography of Mr. Royston, 114; case of puerperal fever, in which the uterus and spleen were principally affected, 117; biography of Menuret, 206; observations on the conical-shaped cornea, by Sir Wm. Adams, 211; account of the reproduction of the vitreous humour of the eye, 215; proposal of a new method of operating for the cataract, by Dr. Lobenstein, 216; cases of peritonitis, by Mr. Bedingfield, 218; account of Gall's and Spurzheim's doctrine of the brain, 290; biography of the Right Reverend Dr. Watson, 379; account of the death of Dr. Rush, 384; on the evolution of heat during the coagulation of the blood, by Dr. Davy, 387; instructions to the apprentices of surgeons, 390; extract from the account of a journey through Egypt, by Mr. Legh, 474; account of the plague in Cyprus, by Dr. Russel, 481; observations respecting the contagion of the yellow fever, by Dr. Haviland, 484; on epilepsy, by Dr. Adams, xxxviii. 39; miscellaneous observations on contagion and contagious diseases, by Dr. Gallup, 45; biographical anecdotes of Mr. John Hunter, by Dr. Adams, 107; adecdotes of Dr. Lettsom, by Mr. Pettigrew, 117; account of the state of medicine in the Tonga Islands, 198; account of the state of medicine in the eastern empire, 203; account of the illness and death of Madame de Stael, by M. Portal, 293; selections on the elephantiasis of Aretæus, 297; account of the fatal accident which happened in the Lead-hill Company's mines, by Mr. Braid, 303; account of Dr. Ingenhouz, by Dr. Garthshore, 377; biographical account of the life and writings of Baron Guyton de Morveau, 382; account of Dr. W. Saunders, 388; account of Dr. C. Combe, 392; of Dr. Wells, 395; of Dr. Werner, 397; history of a case of rupture of the brain, by Dr. Baron, 464; on the internal and external use of the nitro-muriatic acid bath, by Dr. Scott, 466; letters on the health of the metropolis, xxxix. 34; on the muscæ volitantes of nervous persons, by Mr. Ware, 106; on the ophthalmic form of fever, by Dr. Robert Jackson, 111; on the diseases of the skin and genitals, 201; on the pellagra, by Dr. Holland, 210; by Mr. Good and Dr. Parr, 216; on the universality of syphilis, by Erasmus, 219; on the use of sulphurous baths in venereal diseases, &c. 220; on the operation of the salt duties, by Sir Thomas Barnard, 289; a memoir on stuttering, by M. Itard, 375; on the hooping-cough, by Dr. Gamage, 383; on the atmosphere of London, by Mr. Frend, 471; further account of Miss M'Avoy, 274; on the use of caustics and mercurials in syphilis, 477; account of a worm voided from the abdomen of a spider, 478; account of poisoning produced by a decoction of black hellebore, 480; some account of the life of Dr. Woodville, xl. 34; biography of Dr. Rush, 39; on the sounds produced by flames in tubes, 129; account of the discovery of a new alkali, 134; on meteoric stones, 136; on the use of digitalis in epilepsy, 202; on the temperature of the summers that give rise to yellow fever at Philadelphia, 203; case of a child who swallowed a knife, 208; on the population of England, 209; observations on inedia, by Mr. Good, 289; some observations respecting respiration, 291; hints relative to the propagation of the plague, 292; account of the Lunatic Hospital at Avignon, 293; account of a change of colour of the skin of an American Indian, 391; observations on ingurgitation, 392; on the medicinal properties of the lauro-cerasus, 395; on the use of colchicum in gout, 396; on mental alienation, by M. Pinel, 479; on the medicinal measures employed by brute animals, by M. Virey, 486; on the medicinal qualities of the uva ursi, 488.

COLLES, Dr. observations on the cause of the trismus nascentium, xxxix. 497.

COLLIER, Mr. history of a case of ligature of the external iliac artery, xxxvii. 154.

COLLINGWOOD, Dr. cases of diseased vertebræ, xxxvi. 279.

COLLINS, Dr. observations on the medicinal qualities of marine plants, xxxvi. 182.

CONANT, Dr. description of a new mode of extracting stones from the bladder through the urethra, xl. 545.

CONSUMPTION, observations on the influence of different degrees of temperature on, xxxv. 182; observations on, as it occurred in the British army in Spain, xxxvi. 214; observations on its frequency at Malta, xxxvii. 96; remarks on a species of, common in England, 331; observations on the influence of climate on, 360, 456; on the effects of a peculiar regimen in, 395, 456; case of, connected with aneurism of the aorta, 420; observations on the diagnosis between phthisis and chronic hepatitis, xxxviii. 32, 190; observations on the influence of temperature and climate on, 97; observations on the efficacy of prussic acid in the cure of, xxxix. 81; general observations on the origin and treatment of, 499; observations on the vapour of boiling tar in the cure of, 511; on the efficacy of tartar emetic in preventing and curing, xl. 73, 416.

CONTAGION, observations relative to that of the plague, xxxv. 153; observations respecting, made at Malta during the time of the plague, 320; on the communication of dysentery by, 401; observations on the propagation of a fever, by, in the British fleet, 499; various observations respecting, xxxvi. 224; account of a mode of destroying it in letters, by fumigation, 253; some remarks on the distinction between contagion and infection, 371; on the contagion of the yellow-fever, 372; observations respecting that of some species of ophthalmia, 415; remarks on Dr. Chisholm's opinion respecting, xxxvii. 42; remarks on the contagious nature of some epidemics, 41; some cursory remarks on, xxxviii. 17, 18; remarks on the doctrine of, in general, 160, 161; various observations on, 345; account of the chemical analysis of the air in a fever-hospital, where the disease was supposed contagious, xxxix. 274; observations illustrative of that of the plague, 491; observations on the doctrine of that of the yellow-fever, 46, 47; on the agency of contagious miasmata on the system, 54.

CONTAGIOUS DISEASES, account of the plague at Malta, xxxv. 153, 220; account of the leprosy of the Arabians, 210, 311; dysentery regarded amongst, 401; yellow-fever, erroneously so considered, 404; general observations respecting, xxxvi. 224; miscellaneous observations on, 46; observations on several, xxxviii. 160, 161; remarks on the plague, xxxix. 491; observations on the nature of the primitive lesion in, 54; report of M. M. Hallé, Leroux, and Chaussier, respecting the contagious nature of yellow-fever, xl. 82.

CONTUSIONS, remarks on those of the fingers, xxxvii. 341. — *See* Wounds from Gun-shot.

CONVULSIONS, observations on the origin of, from affections of different parts of the nervous system, xxxv. 120; observations on those of children, 240; case of, from ulceration of the brain, 299; from tumors in the cerebrum, 301; observations on the use of emetics in the treatment of some convulsive diseases, xl. 336; observations on puerperal, 403.

COOKE, Mr. account of a substitute for alcohol in anatomical preparations, xxxvii. 180.

COOMBE, Mr. case of hernia cerebri, xxxvi. 449.

COOPER, Mr. A. account of three cases of calculi removed from the urethra without the use of cutting instruments, xxxix. 417; review of his Surgical Essays, xl. 512.

———, Mr. S. observations on lithotomy, xxxix. 76.

COPPER, some observations on the medicinal qualities of, xxxvii. 166; account of a native mass of, xl. 104; experiments on the oxide of, 105.

CORFU, account of the fever prevalent at, in 1815 and 1816, xxxix. 491.

CORK, account of the fever epidemic at, xxxix. 83, 274.

CORPULENCE, observations on, xxxvii. 245, 262, 528.

CORVISART, M. history of a disease of the heart, xxxv. 149.

COUTANCEAU, M. some extracts from his Physiological Observations, xxxvi. 209.

COW-POX, observations on its state in Oxford in 1816, xxxv. 93; some observations respecting its progress, xxxvi. 9; some observations respecting its progress in England, 167; and in Hayti, 169; account of the state of, in France, in 1816, xxxvii. 153; observations relative to its prophylactic power, 255; remarks on the conveyance of it to America, 374; on its antiquity, 531; report of the National Establishment for, in 1817, xxxviii. 174; observations on the anti-variolous power of, xxxix. 160.

CRAMPTON, Dr. Philip, observations on the operations for aneurism, xxxvii. 500; observations on periostitis, xl. 64.

———, Dr. John, history of a case of ulceration and rupture of the stomach, xl. 295; history of a case of hydrocephalic fever, 305.

CRANIOLOGY, observations relative to, in its relation to madness, xxxv. 72; observations relative to, 72, 252; account of the progress of, xxxvii 160; explanatory account of, 290; vindication of, 297; remarks on the unfavourable reception of the system of, 348, 526

CRANIUM, account of a remarkable one in the possession of Mr. Brookes, xxxvi. 254; history of some severe consequences from a slight blow on the head, affecting the, xxxviii. 49.

CRAWFORD, Dr. account of his experimental enquiry into the effects of tonics on the animal fibre, xxxvii. 63.

————, Mr. C. K. history of two cases of a wounded artery in venesection, cured by compression, xxxvi. 331.

————, Mr. J. account of the effects of cubebs in curing gonorrhœa, xxxix. 159.

CRICHTON, Dr. observations on the efficacy of the vapour of tar in relieving some diseases of the lungs, xxxix. 511.

CRITICAL ANALYSIS, of Bichat's Researches on Life and Death, xxxv. 41, 125; of Dr. Carson's Treatise on the Circulation of the Blood, 55; of Mr. Crowe's Chemical Tables, 59; of Dr. A. P. Wilson Phillips's Experiments and Observations on the Relations subsisting between the Nervous and Sanguiferous Systems, 119; of Mr. Rootsey's General Dispensatory, 141; of the forty-fifth number of the Edinburgh Medical Journal, 147, 222; of Mr. Wadd's Cases of diseased Bladder and Testicles, 217; of Dr. Clarke's Commentaries on some of the most important Diseases of Children, 235; of the sixth volume of the Transactions of the Medico-Chirurgical Society, 319, 408; of Schnel's Dissertation on the Upas Antiar Poison, 330; of the forty-sixth number of the Edinburgh Medical Journal, 398, 499; of Mr. Whateley's Observations on the Cure of Wounds and Ulcers of the Legs, 418; of Dr. Parry's Experimental Enquiry into the Nature and Causes of the Arterial Pulse, 478; of Mr. G. N. Hill's Essay on the Prevention and Cure of Insanity, 488; of Mr. Armiger's Rudiments of Anatomy and Physiology, 508; De l'Education Physique de l'Homme, 218; Medico-Chirurgical Transactions, vol. vi. (continued,) a new method of tying arteries in aneurism, amputation, and other surgical operations, by Mr. Lawrence, 227; further observations on the ulceration of the cartilages of the joints, by Mr. Brodie, 229; case of hernia ventriculi, by Mr. Wheelwright, 232; sketch of the medical history of the British armies in the Peninsula of Spain and Portugal during the late campaigns, by Sir James Macgregor, 233; statements of the comparative health of the British navy, from the year 1779 to the year 1814, with proposals for its farther improvement, by Sir Gilbert Blane, Bart. 235; case of the successful treatment of the incontinence of urine consequent to sloughing or ulceration of the bladder, from injury during labour, with observations, by Mr. Barnes, 235; further observations on the ligature of arteries, by Mr. Travers, 235; some experiments on the chemical nature of chyle, &c. by Dr. Marcet, 237; on burns, and the only safe remedy for curing them speedily and without pain, in every stage, by Dr. Dzondi, 239; Edinburgh Medical Journal, No. xlvii., 246; Commentatio de Tracheitide Infantum, &c. auctore J. A. Albers, 306; observations on the treatment of rheumatism by compression, by Dr. Balfour, 318; Dr. Parkinson's Synopsis Nosologiæ, 327; Dr. Reid's Essays on Hypochondriacal and other Nervous Affections, 392; Mr. Bedinfield's Compendium of Medical Practice, 402; Scarpa's memoir on the cutting gorget of Hawkins, 407; Edinburgh Medical Journal, No. xlviii. 410; of the first part of the seventh volume of the Medico-Chirurgical Transactions, 481; of Dr. Stewart's Treatise on Uterine Hæmorrhage, 489; of Dr. Scudamore's Treatise on Gout, 498; of the oracular communication of Æsculapius, 508; of Dr. Parry's Elements of Pathology and Therapeutics, xxxvii. 51, 127; of Dr. Crawford's Enquiry into the Effects of Tonics on the Animal Fibre, 63; Medico-Chirurgical Transactions, vol. vii. part ii. 70, 151, 323, 476; Dr. Griffiths's Essay on Hepatitis, &c. 143; Mr. Clarke's Essay on the Gripes of Horses, 145; of the Observations on the Bills relative to the practice of surgery and midwifery, 146; of Dr. Marcus's treatise on Hooping-cough, 220; of the forty-ninth number of the Edinburgh Medical Journal, 232; of Dr. Dubuisson's treatise on Mental Alienation, 241; of Mr. Wadd's Remarks on Corpulence, 245; of Mr. Salisbury's Practical Botany, 248; of Dr. Armstrong's Practical Illustrations of Typhus, and other febrile diseases, 305; of Dr. Morrison's treatise on Tetanus, 317; of Dr. Lambe's additional reports on the effects of a peculiar regimen in cases of cancer, scrofula, consumption, asthma, and other chronic diseases, 394; of Dr. Halliday's Remarks on Lunatic Asylums, 409; of the Edinburgh Medical Journal, No. l. 413; of Dr. Burrows's statement of the circumstances connected with the Apothecaries' Act, and its Administration, 489; of Mr. Howship's Practical Observations in Surgery and Morbid Anatomy, xxxviii. 48; of Mr. A. C. Hutchison's Practical Observations in Surgery, 62; of Mr.

Parkinson's Essay on the Shaking Palsy, 71; Transactions of the Medical Society of London, vol. i. part ii. 126; Edinburgh Medical Journal, No. li. 133, 245; of Cullen's First Lines of the Practice of Physic, 155; of Mr. Good's Nosology, 210, 306; of Mr. Marshall's remarks on arsenic, 240; of Mr. Curtis's treatise on the Physiology and Diseases of the Ear, 326; of M. Alibert's Elements of Therapeutics, 330; of M. Guilbert's Essay on Gout, 331; of the Picture of the London College of Physicians, 403; Edinburgh Medical Journal, No. lii. 406; Medico-Chirurgical Transactions, vol. viii. part i. 411; of Dr. Wistar's System of Anatomy, 414; of Dr. Hendriksz's Critical Description of the principal Surgical Operations performed in the Academical Hospital at Groningen, from 1810 to 1815, 421; of Mr. Accum's Chemical Amusement, 422; of Mr. Charles Bell's Surgical Observations, part i. 476; of Dr. Mills's Morbid Anatomy of the Brain in Typhus Fever, 491; of Mr. Pring's View of the Relations of the Nervous System, 495; of Mr. Dunn's Suggestions for the Relief of the Sick-Poor, 511; of Dr. Jackson's Sketch of the History and Cure of Febrile Diseases, xxxix. 49; of Dr. De Sanctis's Lusus Naturæ Descriptus, &c. 67; of the Medico-Chirurgical Transactions, vol. viii. part i. 71; of Dr. Renwick's Narrative of the Case of Miss Margaret M'Avoy, with an account of some optical experiments connected with it, 114; of Mr. Sandars's Hints to Credulity; or, an Examination of the Pretensions of Miss M'Avoy, occasioned by Dr. Renwick's Narrative of her case, ibid.; of Sir William Adams's Practical Inquiry into the Causes of the frequent failure of the Operations of Depression, and of the Extraction of the Cataract, as usually performed, 126; of Dr. Mensert's treatise on the Operation of Keratonyxis, 134; of Mr. Wright's essay on the Human Ear, 139; of Mr. Bailey's Observations relative to the use of belladonna in painful disorders of the head and face, 141; Edinburgh Medical Journal, No. liii. 155; of Mr. Johnson's Treatise on the Influence of the Atmosphere on the Health and Functions of the Human Frame, 247; of Mr. William Wadd's Cases in Surgery, part ii. 304; of Dr. John Vetch's observations relative to the treatment, by Sir William Adams, of the Ophthalmic Cases of the Army, 308; of Dr. Marcet's essay on the Chemical History and Medical Treatment of Calculous Disorders, 313; of the Medico-Chirurgical Transactions, vol. viii. part ii. 322; of Dr. Schmidt's Commentary on the Pathology of the Spleen, 238; of Mr. Yeatman's remarks on the medical care of Parochial Poor, 331; of Sir Everard Home's Paper on the passage of the ovum from the ovarium into the uterus, 395; of the Dublin Hospital Reports, vol. i. 482; of Dr. Armstrong's Practical Illustrations of the Scarlet Fever, Measles, Pulmonary Consumption, and Chronic Diseases, 499; of Dr Dewar's Account of an Epidemic Small pox, which occurred in Cupar in Fife, in the spring of 1817, and the degree of protecting influence which vaccination afforded, 405; of Mr. C. Bell's Surgical Observations, xl. 48, 139, 222; of the Dublin Hospital Reports, continued, 62; of Dr. Williams's observations on Dr. Wilson's remedy for gout, 146; of Mr. Hennen's Observations on Military Surgery, 154, 214; of Mr. Blackadder's Observations on Phagedæna Gangrænosa, 226; of Dr. Wilson's Observations on Morbid Sympathies, 242, 404; of the association of the College of Physicians in Ireland, 222, 398; of the Edinburgh Medical and Surgical Journal, No. lv. 313; of Mr. Jardine's essay to improve the instruments for some surgical operations, 324; of Mr. Newnham's essay on Inversion of the Uterus, 325; of the Transactions of the Physico-Medical Society of New-York, 331, 425, 527; of M. Magendie's Researches into the Causes, Symptoms, and Treatment, of Gravel, 338; of M. Lallemand's Thesis, 343, 431, 531; of Dr. Balfour's Observations on the use of emetic tartar, 410; of Mr. Dickenson's Treatise on Burns and Scalds, 417; of Dr. Callanan's Treatise on Typhous Fever, 491; of Messrs. Cooper's and Travers's Surgical Essays, 502.

CROFT, Sir R. some account of the life of, xxxix. 259.

CROSS, Dr. history of a case of paralysis, xxxvi. 194; description of two cases of tetanus, xxxvii 19.

———, Mr. observations on the ligature of arteries, xxxvii. 521.

CROUP, case, showing the utility of copious bleeding in, xxxv. 79; case of, in which bronchotomy was successfully performed, 417; account of a general treatise on, 306; observations on the efficacy of the sulphuret of potash, in the cure of, 373; observations on the treatment of, by henbane, xxxvii. 164; observations on a variety of, xl. 335.

CROWE, Mr. account of his Chemical Chart, xxxv. 59.

CROWFOOT, Mr. observations on the poisonous effects of arsenic, xxxviii. 446; xxxix. 336.

CRUVELHIER, M. notice of his work on Pathological Anatomy, xxxvii. 170, 519.

CRYSTALLIZATION, experiments and observations on the laws of, xl. 92.

CUBEBS, observations on the efficacy of, in the cure of gonorrhœa, xxxix. 159.

CULLEN, observations on the Practice of Medicine of, xxxviii. 155.

CUPPING, account of the operation of, from an early English work on Surgery, xxxv. 383; account of a new mode of, xxxvi. 83.

CURRIE, Dr. remarks on his precepts for the use of cold affusion, xxxvii. 38.

CURRY, Dr. remarks on the doctrine respecting the secretion of pus, xxxviii. 196; xxxix. 17, 79, 383.

CURTIS, Mr. observations respecting his work on diseases of the ear, xxxvii. 253; xxxviii. 327, 429.

CUSACK, Dr. account of the removal of a large tumor from the neck, xl. 62.

CUTANEOUS DISEASES, observations respecting their connection with parasitical insects, xxxv. 466; observations on the efficacy of sulphurous fumigation in, xxxvii. 85; remarks on the arrangement of, 419; on the method of curing them by nitro-muriatic baths, 527; some remarks on the difficulties attendant on the arrangement of, xxxviii. 19, 20; account of those resembling syphilis, lately epidemic in Canada, xxxix. 209.

CYNANCHE MALIGNA, observations on the treatment of, xxxvii. 68.

CYNANCHE PAROTIDEA, observations on the treatment of, xxxv. 424.

CYPRUS, account of the plague at, xxxvii. 481.

D.

DALRYMPLE, Mr. a case of aneurism, by anastomosis, cured by tying the carotid artery, xxxv. 411.

DARWIN, Dr. Erasmus, remarks on some parts of his medical doctrine, xxxviii. 6, 7, 8.

DAVIES, Rev. H. observations on the sea long-worm, xxxvi. 207.

DAVIS, Dr. description of his obstetrical instruments, xxxviii. 91.

DAVY, Sir H. extracts from his observations on chlorine, xxxv. 111.

———, Mr. Edmund, an account of a mode of making good bread from damaged flour, xxxvii. 160; observations on the state of the air in the fever-hospital at Cork, xxxviii. 274.

———, Dr. John, observations on the heat evolved during the coagulation of the blood, xxxvii. 338; chemical examination of some substances used in Ceylon as remedies against the bites of venomous serpents, xxxix. 284.

DEAFNESS, observations on the means of relieving some species of, xxxvi. 36, 426; xxxvii. 258.

DEATH, account of Bichat's Researches on, xxxv. 41; experiments relating to the causing of, by destruction of different parts of the nervous system, 119, 126; observations on the different ways in which it takes place, 317; history of cases of voluntary similation of, xxxvi. 394; observations on the appearance of the blood-vessels after, xxxvii. 55.

DEBILITY, observations on, in fever, xxxv. 355; on the erroneous view in which it has been regarded in fever, xxxvi. 156; general observations on, xxxviii. 6, 7.

DELIRIUM, observations on it, as connected with mania, xxxvii. 243; some remarks on the management of it, when present in typhus fever, xxxix. 483; observations on its importance in continued fever, 49.

DEMERARA, medical topography of, xxxvii. 317.

DENMARK, Dr. observations on the Mediterranean fever, xxxvi. 155.

DENTITION, observations on the process of, xxxvi. 221.

DEPLETION, observations on, in the treatment of febrile diseases, xxxv. 355; observations on the use of, in the cure of fever, 407; observations on the use of, in the treatment of fever, 368, 369, 370; remarks on the general effects of, xxxvii. 12; on its use in typhus, 316; on its use in diabetes, 107.

DESGENETTES, M. his opinion respecting the contagious nature of yellow fever, xxxvii. 41.

DEWAR, Dr. remarks on the preservation of lime-water, xxxvi. 419; on the treatment of sinuous ulcers, 513; account of small-pox, as it appeared as an epidemic at Cupar in Fife, xxxix. 509.

DIABETES, history of a case of, in which opium was apparently beneficial, xxxvi. 26; distinction between the urine in that affection and in the state of health, 31; cases of, in which bleeding was beneficial, 115; facts and observations respecting, xxxvii. 186; observations on the state of the urine in, 189; observations respecting the composition of the urine in, xxxix. 322; observations respecting the use of magnesia in the treatment of, 366.

DIAGNOSIS, on the difficulties attending that of diseases of the brain, xxxv. 295; of the effects of arsenic, 338; various observations relative to, xxxvi. 417; of inflammation of the large intestines, 418; on that between phthisis pulmonalis and chronic hepatitis, xxxviii. 32, 190; on the value of the signs presented by the appearance of the tongue in fever, xl. 312.

DIAMOND, observations on the natural history of the, xxxix. 432.

DIARRHŒA, observations on the use of muriatic acid in that of children, xxxv. 73; case of severe, accompanied with discharge of semi-organized mucous matter, xxxviii. 193.

DICKINSON, Mr. Nodes, review of his work on Burns and Scalds, xl. 417.

DICKSON, Dr. J. H. observations on the utility of blood-letting and purgatives, in a fever which prevailed in the Russian fleet, xxxv. 499; remarks on the review of those observations, xxxvi. 130; some remarks on the treatment of fever, 371; on the causes of the tropical endemic, or yellow-fever, xxxvii. 233; observations on tetanus, 326.

DIEBY, Dr. history of a remarkable case of obesity, xxxvii. 528.

DIET, observations on the use of spare, in fever, xxxv. 355; effects of a peculiar, in the cure of asthma, xxxvii. 394; observations on the use of a certain, in consumption, 395.

DIGESTION, some observations respecting it, xxxv. 55, 331; xxxix. 2.—*See* STOMACH.

DIGITALIS PURPUREA, observations on the peculiar advantages of certain modes of administering it, xxxviii. 339; observations on the medical effects of, xxxix. 91, 94; observations on its use, as a local application in scrofula, xl. 119; its efficacy in the cure of epilepsy, 204.

DISCOVERIES, account of that of the descent of the testicle, by John Hunter, xxxv. 109; account of those relating to chlorine and iodine, 111; account of some, respecting the relations subsisting between the nervous and sanguiferous systems, 42, 119, 127.

DISEASES, reports of, for London, xxxv. 79, 168, 254, 342, 432, 518; xxxvi. 86, 174, 261, 350, 439, 526; xxxvii. 174, 263, 351, 436, 532; xxxviii. 83, 176, 264, 350, 438, 520; xxxix. 86, 176, 262, 478, 526; xl. 85, 176, 261, 357, 455, 550.

——————, observations on those especially prevalent in different classes of society, xxxv. 328; observations respecting the influence of the air on the character of, 417; observations on predisposition to, 495; xxxvi. 501; remarks on those reputed contagious, xxxvii. 50; (*See* CONTAGIOUS DISEASES:) remarks on constitutional, 517; remarks on the common origin of, 137; remarks on those of dumb persons, 258; observations on chronic, xxxix. 169, 499.

DISSECTIONS, of the body of a person who died from obstruction of the intestines by a cocoa-nut, xxxv. 103; of a case of malformation of the heart, 148; in three cases of inflammation of the heart, 223; of some cases of disease of the brain, 297, 298, 299, 300, 303, 304, 307, 308, 309, 310; in a case of inguinal aneurism, where the iliac artery was tied, 380; in cases of dysentery, 402; in a case of tetanus, 410; of the body of a girl, in which a tumor containing parts of a fœtus was found in the abdomen, 412; in a case of enteritis, with preternatural formation of the ilium, 436; in some cases of inflammation of the larynx, xxxvi. 61; in some cases of diseases of the joints, 232; in a case of hernia of the stomach through an opening in the diaphragm, ibid.; in a case of mortification of the appendix coeci vermiformis, from the presence of a tooth, 249; in two cases of humoral asthma, 332; in a case of hydrocephalus, 388; in a case of ulceration of the pharynx, with inflammation of the larynx, 403; in a case of ulceration of the larynx, 404; in a case of ulceration of the trachea and larynx, 405; in cases of inflammation of the larynx, 422; in a case of hernia cerebri, 449; in two cases of hydrophobia, 517; in a case of rupture of the ilium, 518; of the body of Professor de Saussure, xxxvii. 73; of the body of Mr. Royston, 116; in a case of puerperal fever, 120; in a case of general transformation of the thoracic and abdominal viscera, 157; in a case of extra-uterine pregnancy, 196; in a disease of the ovarium, 197; in some cases of peritonitis, 219, in cases of hooping-cough, 223; in a case of disease of the heart, 323; in a case of tetanus, 330; in a case of polypus and enlargement of the heart, 416; in a case of disease of the brain, 428; in a case of enteritis, 446; in a case where the femoral artery had been tied, 505; in a case of extra-uterine gestation, 510; in a case of disease of the cranium and brain, xxxviii. 49; in inflammation of the trachea, 52, 53; in inflammation of the lungs, 55; in a disease of the ovarium, 58; in a case of internal strangulation of the small intestines, 169; in a case of aneurism of the heart, 188; in a case of severe diarrhœa, 193; in a case where the internal iliac artery had been tied, 267; of the brain, in typhous fever, 491; of the body of the Princess Charlotte of Wales, 513; in a case of ossification of the heart, xxxix. 7; in a case of disease of the heart, 9; in several cases of fever, 50, 51, 60, 61, 62, 63, 487; in a case of rupture of the stomach, 159; in a case of spina bifida, 184; in a case of inflammation of the uterus, 344; in cases of hooping-cough, 384, 385, 391, 392; in a case of gun-shot wound of the heart, 404; in several diseases of the heart, 420, 421, 422, 423; in cases where ligatures had been applied to large arteries, 427, 428; in a case of poisoning by black hellebore, 481; in a case of jaundice, 496; in a case of unusual distribution of the abdominal and thoracic viscera, xl. 339; in a case of rupture of the colon, 15; in cases of tetanus, from disease of the spinal marrow, 259, 303; in a case of ulceration and rupture of the stomach, 295; in a case of rupture of the stomach from vomiting, 532; of the bodies of two habitual drunkards, 299; in a case of extra-uterine gravidity, 343; in a case of rupture of the heart, 425; of an acephalous fœtus, 346; in a case of suppuration of the liver, 298; in a case of severe disease of the brain, 447.

DISTENSION, *vascular*, observations on excess of, as connected with diseases, xxxviii. 373.

DONNELL, Mr. account of the juridical enquiry respecting, xxxvii. 429.

DOUGHTY, review of his work on yellow-fever, xxxvi. 140.

DOUGLASS, Mr. history of a case of wounded bladder, xxxviii. 346.

DOVAR, Dr. remarks on his mode of using opium, xxxix. 448.

DRAPER, Mr. remarks on some of the remedies used in fevers, xxxvii. 240.

DROPSY, of the belly, case of, 434; observations on the use of the ballota in the cure of, xxxvi. 346; some clinical observations on, 515; observations on the nature and causes of the effusions in, xxxvii. 129; pathological observations on, 128; observations respecting its connexion with tabes mesenterica, xxxix. 93; observations on its origin, from conversion of diseases from the mucous to the serous membranes, 498; observations on the use of bleeding, in some cases of, 411; observations on its connexion with inflammation of the lungs, xl. 305; cases of ovarian, 306.

DUBLIN, account of the institution at, for administering aid to the sick poor, xxxv. 328; account of the epidemic fever of, xxxix. 482.

DUBUISSON, M. observations on mental alienation, xxxvii. 341.

DUCHATEAU, M. account of a case of disease of the heart, xxxix. 9.

DUMERIL, Mr. observations on the duties and acquirements of medical students, xxxvii. 259.

DUNCAN, Dr. jun. case of disease of the heart, xxxv. 225.

———, Mr. Thomas, case of carotid aneurism, xxxv. 147.

DUNDAS, Sir David, his Hunterian oration, xxxix. 253.

DUNN, Mr. case of extirpation of part of the bones of the ankle-joint, xxxvii. 522; suggestion for the relief of the sick poor, xxxviii. 511; observations on puerperal fever, xxxv. 222.

DUNNING, Mr. account of a case of cancer treated by pressure, xxxvii. 354.

DUPLAN, Mr. history of a remarkable case of strangulated hernia, xl. 543.

DUVAL, Mr. observations on the preservation of the teeth, xxxvi. 255.

DWARF, description of a female, xxxix. 258.

DYSENTERY, observations on the causes and treatment of, xxxv. 399, 401, 456; xxxvi. 130; xxxvii. 319, 532; xxxix. 154.

DYSPEPSIA, observations on the causes and treatment of, xxxvii. 271, 324.

DZONDI, Dr. observations on the treatment of burns, xxxvii. 239.

### E.

EAR, observations on the puncture of the membrane of the tympanum, xxxv. 253; case of disease of, connected with lesion of the brain, 298; notice of a treatise on the diseases of the, xxxviii. 326.

EARLE, Mr. H. observations on the use of tobacco in retention of urine, xxxv. 408.

EARTH-EATERS, account of the, of Africa, xxxviii. 79.

EDGELL, Mr. observations on the practice of midwifery, xxxv. 350; case of placental presentation, 462; on the use of turpentine in puerperal peritonitis, xxxviii. 447.

EDINBURGH, account of the mode of conducting medical education at, xxxix. 192.

EDWARDS, Mr. account of a case of placental presentation, xxxix. 443.

EFFLUVIA, marsh, considered as the cause of yellow-fever, xxxv. 404; observations respecting its influence, in the production of dysentery, xxxvi. 138; observations on its existence as a common cause of fever in England, 246; observations on those of the marshes of Italy, xxxviii. 345.

EGG, account of a singular formation in an, xxxviii. 173.

EGYPT, contributions to the medical topography of, xxxvii. 474, 477.

ELECTRICITY, observations on the identity of the electric and nervous fluid, xxxv. 104.

ELEPHANTIASIS, description of, xxxv. 210, 311, 393; xxxviii. 297; xxxix. 216, 307, 26; history of a case of, xl. 353.

EMBDEN, Dr. Von, account of Dr. Marcus's treatise on hooping-cough, xxxvii. 220; reply to the reviewer of the Continental Medical Repertory, xxxviii. 426.

EMETICS, account of those produced in guiana, xxxvi. 343; observations on the use of, in gout, 505; observations on their efficacy, in hooping-cough, xxxvii. 328; observations respecting their use, in typhous fever, 310; observations respecting their use in spasmodic diseases, xl. 336.

ENDEMICS, observations on tropical, xxxvii. 233; remarks on the term, xxxix. 43.

ENEMAS, description of a new machine for administering them, xl. 200.

ENEURESIS, case of, cured by the use of bougies, xxxv. 411.

EPIDEMICS, observations respecting those prevalent in America, xxxviii. 165; various observations respecting, xxxix. 44; observations on their connexion with certain poisons, 36; account of the fever epidemic in Dublin, xl. 307, 378, 398; of that in Cork, 491. —*See* CONTAGIOUS DISEASES.

EPIGASTRIUM, observations on pulsation in the, xl. 529.

EPILEPSY, general observations on the nature and treatment of, xxxviii. 39; remarks on its hereditary nature, xxxvii. 137; on the efficacy of the nitrate of silver, in the cure of, 166; case arising from disease of the spine, 262.

ERYSIPELAS, case of metastasic, xxxv. 302; observations on the treatment of, xxxvii. 314; observations on its occurrence in hospitals, xxxviii. 66.

ESQUIROL, M. observations on insanity, xl. 542.

ESTLIN, Mr. observations on diseased spine, xxxviii. 345.

EXERCISE, observations on its use by the ancients, xxxvii. 346.

EXOSTOSIS, observations on the origin and treatment of, xl. 516; histories of two peculiar cases of, xxxviii. 355; xl. 364.

EXPERIMENTS, account of Bichat's respecting life and death, xxxv. 41; on the cure of syphilis without mercury, 73; account of those relating to the discoveries of chlorine and iodine, 111; account of Dr. A. P. Wilson Phillips's, on the relations between the nervous and sanguiferous systems, 119, 127; observations respecting some made on animals, to ascertain the cause of their heat, 291; remarks on some, executed to determine the cause of the action of the heart, 317; account of some, with the upas antia, 330; on the nervous influence in secretion, 331; on the connexion of the circulation of the mother with that of the fœtus, 134; to determine the relation of galvanism and the nervous influence, 423; observations respecting the mode of conducting them in animals, in physiological researches, 460; to determine the cause of the arterial pulse, 478; with the spurious angustura, xxxvi. 162; on the nature of chyle, 237; on the crystallization of pure lime, 255; with the ergot rye on animals, 378; respecting the contagious nature of some species of ophthalmia, 415; respecting the generation of animal heat, 486; on the urine of gouty patients, 503; with the venom of the viper, 522; on the effects of tonics on the animal fibre, xxxvii. 65, 67; on the production of new arterious vessels, 100; observations on those made on living animals, 110, 168; on the means of correcting musty corn, 156, 161, 429; remarks on the mode of conducting, 270; on animal heat, 283; on the qualities of different nutritious substances, 343; on the evolution of heat during the coagulation of the blood, 388; on the venom of the viper, 434; on the obliteration of the arteries, 500; on the effects of ligatures, in amputation of the thigh, 521; on fossil bones, 522; on flame, 524; on inoculating cows with variolous matter, 526; on intestinal gas, 529; directions for various chemical, xxxviii. 422; on the air, at the fever-hospitals at Cork, xxxix. 274; on refuse salt, 289; on the use of the spleen, 330; on spectra, 357; on urine, 455, 332; on the duration of utero-gestation in different animals, 6; with morphine, xl. 275; on the influence of the qualities of the aliment on those of the urine, 340; on the digestive functions of the stomach, 531.—*See* CHEMISTRY.

EYE, observations on affections of the, connected with disease of the brain, xxxv. 296, 299, 301, 302, 303, 304, 310; on the peculiar state of, in children, suffering inflammation of the membranes of the brain, 339; cases of the formation of an artificial pupil, xxxvi. 49; observations on the causes which alter the size of the pupil, 89; some observations on the mechanism and functions of those of fishes, 166; observations on some diseases of the, connected with rheumatism, 202; observations on the causes which enlarge the pupil of the, 265; observations on its sympathy with the intestines, 346; observations on rheumatic inflammation of the, 382; observations respecting some contagious inflammation of the, 415; account of a painful affection of the appendages of the, 442; account of some new instruments for operations on the, xxxvii. 81; account of the reproduction of the vitreous humour in the, 215, 455; account of a new mode of operating for the cataract in the, 216; observations on wounds of the, 257; observations on some measures in the treatment of diseases of the, 273; on the connexion between the, 282; case of imperfection of the, 331; observations on the operation for artificial pupil in the, 496; observations on amaurosis, 392; on amphyopy, 280; account of the effects of severe cold on the, 170; observations on the use of moxa, in some affections of the, ibid.; case of fungous tumor of the right, xxxviii. 179; observations on a species of disease of the, especially affecting sickly children, 185; some observations respecting the size of the pupil of the, under different circumstances, 283; observations on opacity of the cornea, xxxix. 310; observations on diseases of, causing the phenomena of muscæ volitantes, 103, 105; remarks on Sir William Adams's treatment of the disease of, in the army, 308; observations on the structure and functions of the retina, xl. 178; observations on the morbid states of the, causing the phenomenon of muscæ volitantes, 182; observations on the refractive powers of different parts of the, 179; observations on venereal inflammation of the iris, 504; remarks on the opinions respecting the muscular power of the fibres of the crystalline lens, 177; on the structure and functions of the retina, 179; on the refractive powers of different parts of the, ibid.

### F.

FACE, observations on the use of belladonna, in painful affections of the, xxxix. 191; case of worms in the sinuses of the, xxxv. 252.

FARRADAY, Mr. observations on the sounds of flames in tubes, xl. 129.

FAT, experiments and observations on its chemical properties, xl. 118.

FEMALES, remarks on some of the diseases to which they are expressly subject, xxxv. 323; observations on the diseases of the Caribbean, 476; account of a fatal affection of the pudendum of, in children, xxxvi. 482; advice to, at certain periods of life, xxxviii. 520.

FERGUSON, Dr. an enquiry into the origin and nature of the yellow-fever of the West Indies, xxxix. 149; observations on the prevention of typhous fever, xl. 21.

FEVER, observations respecting the utility of ablution with diluted sulphuric acid, in typhus, xxxv. 73; observations on the utility of copious bleeding in, 79; observations on blueness of the nose in typhus, 156; observations on the remittent, of infants, 329; observations on, originating in inflammation of the cerebral membranes, 339; observations on the treatment resorted to in typhus, in different ages, 353; remarks on the propriety of attending to the state of the fluids, in the theory of, 354; on the appropriate treatment of, 356; observations on the nature of typhus, 404; remarks on the use of calomel, in the cure of, 407; on the use of blood-letting, in, as it occurred in the British fleet, 499; observations on that prevalent at Gibraltar in 1813, and especially on the use of blood-letting in it, 505; observations on the Mediterranean, 155; remarks on the question respecting its local or general origin, 155; observations on the utility of blood-letting, in, 246; observations on the depletory treatment of, 368; observations on some of the remedies used in the treatment of, xxxvii. 240; observations on the occurrence of blueness of the nose in typhus, 305; observations on the term, 305; observations on the use of emetics and purgatives, in the treatment of, 310; practical illustrations of typhus, and other species of, 305; observations on the open forms of, 309; on the congestive form of, 315; on the treatment of inflammatory typhus, 312; on the treatment of congestive typhus, 315; cautions respecting depletion, in the treatment of, 316; remarks on the prevalence of typhus, 436; account of, as it prevailed at Ipswich in 1816, 451; observations on, as it prevailed at Cambridge in 1816, 484; observations in favour of the cold affusion in, 311; remarks on the modern improvement in the arrangement of the different species of, xxxviii. 16, 17; remarks on the local origin of, 158, 159, 160; observations on the use of cold application to the head, in typhus, 278; observations on typhus fever, especially shewing its origin from local inflammation, illustrated with cases, 406; observations respecting the efficacy of cold air, &c. in, 433; observations illustrative of its origin from inflammation of the brain, 491; account of a general treatise on, xxxix. 45; observations respecting its local origin, 371; observations on the mercurial plan of treating, 57; remarks respecting an ophthalmic form of, 111; remarks on, continued, 362; observations respecting that recently epidemic in Ireland, 483, 408; remarks on remittent, 491; on the influence of the heating regimen in, 485; observations on the diurnal exacerbations and remissions of, 48; observations on the local sufferings in, ibid.; on the termination of, especially by crisis, ibid.; on the state of the brain and intestinal viscera, in, 60, 61; observations on the efficacy of copious blood-letting in, 55; on the use of cold water in, 56; on the use of exercise in, 57; cautions respecting the use of blood-letting in, 485; on the use of purgatives in fever, 486; observations on the state of the intestinal canal in relapses in, 487; some observations on the causes of, xl. 406; some observations on the doctrine of critical days in, 279; observations on that epidemic in Dublin, 307, 398, 399; remarks on the unfavourable symptoms in, 312; on the crisis of, ibid.; remarks on the diagnostic signs presented by the tongue in, ibid.; case, in which signs of inflammation were suddenly developed at an advanced stage of, in a mild case of, 498; account of that epidemic in Cork, 491; remarks on the prevention of contagious typhus, 21; observations on its connexion with disease of the spinal marrow, 321; on its division into simple, inflammatory, and congestive, 493, 499; observations on its connexion with hydrocephalus, 306.

FEVER, *puerperal*, case of, accompanied with dropsy, succeeded by phlegmasia dolens, gastritis, and spontaneous hydrophobia, xxxv. 12; account of a mode of treating it, by injections into the uterus, xxxvi. 1; cases of, in which injections into the uterus were employed, apparently with benefit, 272; observations on its nature and origin, 512; case of, in which the uterus and spleen were particularly diseased, xxxvii. 117; queries respecting a case of, 357; observations on the use of oil of turpentine in, xxxviii. 447; case of, relieved by glysters of warm milk, xxxix. 513.

———, *scarlet*, case of, followed by anchylosis of several joints, xxxvii. 277; observations on the varieties of, from the same infection, 177; case of anchylosis of the joints, consequent on, 277; review of a general treatise on, xxxix. 499; observations on the nature and forms of, xl. 477.

———, *yellow*, remarks on the benefit of the antiphlogistic method of treatment in, xxxv. 366; observations on the nature and origin of, considered to be produced by marsh miasma, 404; observations on, as it affects the Caribbeans, 476; Dr. Pym's observations, to shew that it attacks the human system but once, 507; observations on its nature and origin, especially as relating to marsh and effluvia, xxxvi. 141; observations on the liability of a person to a second attack of, 330; observations respecting its contagious nature, 372; remarks on its contagious nature, xxxvii. 38; observations on its nature and origin, 233, 207; observations on the mercurial treatment of, xxxviii. 487; various observations on, especially relating to the black vomit in, xxxix. 50; enquiry respecting its nature and origin, 149; observations respecting it, from atmospheric influence, 36; observations on the doctrine of its contagious nature, 49; report made by M. M. Hallé, Leroux, and Chaussier, respecting its contagious nature, xl. 82; observations respecting the temperature of the season which gave rise to it at Philadelphia, 205.

FIRE-BALLS, observations on the mode of observing them, xxxix. 341.

FISKE, Dr. cases of inflammation of the larynx, and of erysipelas, xxxv. 346.

FLAME, observations on the nature of, xxxvii. 524.

FLOCKBERG, account of the waters of, xxxvii. 267.

FŒTUS, experiments to ascertain the connexion of the circulation of the, with that of the mother, xxxv. 134; case of parts of one being found in a tumor within the abdomen of a girl two years and a half old, 411; case of the preservation of the life of one, born within the fifth month of uterine gestation, 424; observations on deformity of the, xxxvi. 219; case of one contained in the fallopian tube, xxxvii. 510; observations on the effects of the venereal disease in the mother, on the, 335; remarks respecting acephalous and akerious, 7; account of one with two heads and two hearts, xxxviii. 338; observations and reflections respecting the destruction of the brain in the fœtus, xl. 431; remarkable instances of that phenomenon, 346, 434; remarks on the nature of the life of the, 435; remarks on the causes of the destruction of the spinal marrow, in the, 432.

FONTANA, Abbé, remarks on some of his opinions respecting the poison of the viper, xxxvii. 434.

FOOD, account of long abstinence from, in a pig, xxxv. 511; observations on the necessity of azote, in that of some animals, xxxvii. 343; accounts of several instances of abstinence from, xl. 289; observations on the influence of azoted and non-azoted aliments on the nature of the urine, 340; observations respecting ingurgitation of, 393.

FOOT, observations on amputation of the, xxxv. 74; xxxvii. 354.—*See* Amputation.

FORSTER, Mr. W. some craniological observations in relation to insanity, xxxv. 72.

FOURNIER, Mr. case of strangulated hernia, xl. 543.

FOX, Mr. observations on the management of the teeth, xxxvii. 443.

FRACTURES, observations on those of the ribs, xxxviii. 475; observations respecting the union of, xl. 538, 427, 428; remarks on the treatment of compound, from gun-shot wounds, 169.

FRANCIS, Dr. case of enteritis, accompanied with a preternatural formation of the ilium, xxxv. 436.

FRANCK, Dr. of Vienna, observations on the use of urtication in paralysis, xxxv. 73; case of worms in the sinuses of the face, 252; observations on the acid of sugar, in scurvy, 338; observations on tetanus, xl. 259.

FREND, Mr. observations on the atmosphere of London, xxxix. 471.

FROG, some observations on the growth of the, xxxix. 432.

FUGE, Mr. case of gun-shot wound of the heart, xxxix. 404.

FUMIGATIONS, sulphurous, observations on their use, in various cutaneous disorders, xxxvii. 85.

FUNCTIONS, in general, experiments relating to the influence of different parts of the nervous system on the, xxxv. 119; some observations on the, xxxvi. 120.

FUNGUS HÆMATODES, case of, with observations, xxxviii. 179, 181; xxxix. 155.—*See* Tumors.

G.

GAITSKELL, Mr. observations on croup, xxxviii. 128.

GALES, M. observations on the use of sulphurous fumigations, xxxvii. 85.

GALL, Dr. some remarks on his doctrine, xxxvii. 135, 290.—*See* Brain, *and* Craniology.

GALLS, observations on their effects on the animal fibre, xxxvii. 67; observations on the essential oil of, xl. 113.

GALLUP, Dr. observations on infectious and contagious diseases, xxxviii. 46.

GALVANISM, observations on its influence on the animal economy, xxxv. 332, 423; xxxvi. 412; observations on its use in the cure of diseases, 165.

GAMAGE, Dr. observations on hooping-cough, xxxix. 14, 203.

GANGLIONIC SYSTEM, observations on the functions dependant on the nervous influence of the, xxxv. 42, 120; anatomical description of the centres of the, 139; remarks on the influence of the, on the action of the heart, 319; observations on the origin and properties of the powers of the, 332; facts tending to prove that it is alone necessary for the direction of the functions of organic life, xl. 346.

GANGRENE, observations relative to the nature and causes of the hospital, xxxv. 20; observations on, especially as it affects the genitals, xxxix. 203; observations respecting the origin of that of hospitals, 14; further observations and remarks on hospital gangrene, 216, 219, 226, 239; observations on its origin from gun-shot wounds, 220.

GARDANNE, M. Avis aux Femmes, &c. xxxvii. 520.

GARDEN, Mr. extracts from his lectures on Zoonomia, xxxviii. 7.

GAS, observations on intestinal, xxxvii. 529; observations on that of bitumen, 339; on the benefits of baths of, 527.

GASOMETER, description of a new one, xl. 92.

GAYE, Mr. remarkable case of ascites, xxxv. 434.

GAY-LUSSAC, Mr. observations on the crystallization of lime, xxxvi. 255.

GECKO, observations on the structure of the feet of the, xxxv. 333.

GEOGHEGAN, Mr. history of a case of abscess in the liver, xl. 306.

GENERATION, observations relative to that of worms, xxxv. 335; case of parts of a fœtus being found in a tumor in the abdomen of a girl two years and a half old, 411; remarks on the doctrine of moral influence, in the act of, xxxvi. 219.

GENITALS, account of the structure of those of the female Hottentot, xxxvi. 466; history of a fatal affection of the pudendum of female children, 482; cases of malformation of the, xxxix. 67, 519; some remarks on diseases of the, 201, 305, 306.

GIBBON, Mr. J. case of premature parturition artificially excited, xxxvii. 15.

GIBRALTAR, account of the fever prevalent at, in 1803, xxxv. 505; some observations on the influence of the climate of, on the human body, xxxvii. 461, 465.

GINGER, observations on its use, in gout, xxxvii. 342.

GIRDLESTONE, Dr. case, in which apoplectic symptoms accompanied the passage of a gall-stone through the biliary duct, xxxix. 441; case of exomphalos, with stricture and rupture of the colon, xl. 13.

GLEN, Mr. account of the poisonous effects of the agaricus campanulatus, xxxvi. 451.

GLOVER, Rev. T. account of Miss M'Avoy, xxxviii. 366.

GODEN, Mr. observations on hydrophobia, xxvi. 74.

GOELIS, Dr. observations on the inflammation of the membranes of the brain and spinal marrow, xxxv. 339; case of paralysis of the heart, xxxviii. 186

GOLD, observations on its medicinal properties, xxxix. 515.

GOMES, Dr. account of his mode of fumigating infected letters, xxxvi. 252, 254.

GONORRHŒA, observations on the use of cubebs, as a remedy for, xxxix. 159; a new injection for, 176; observations on the opinion respecting the existence of ulceration of the urethra, in, 286.

GOOD, Mr. review of his System of Nosology, xxxviii. 210, 360; his description of pellagra and elephantiasis, xxxix. 216; observations on inedia, xl. 289.

GOODISON, Dr. account of the plague at Corfu, xxxix. 491.

GOODLAD, Mr. account of the removal of a large tumor from the face and neck, xxxvii. 152.

GORDON, Dr. observations respecting his remarks on craniology, xxxvii. 160, 290; observations on the coagulation of the blood, 287; case of injury of the spinal cord, xxxviii. 406.

GORGET, observations on the use of the, xxxvi. 408.

GOUT, observations on the danger of suppressing it, xxxv. 268, 269; remarks on the more favourable manner in which the cooling treatment of, has been regarded, since the publication of the work of Dr. Kinglake, 366; reference to Sydenham's opinions on the use of bleeding, in, 414; observations on the treatment of, by compression and percussion, 420; review of a treatise on, 498; observations on the use of ginger, in, xxxvii. 342; observations on the comparative advantages of the cooling and heating treatment of, xxxviii. 383; general observations on the nature, origin, and treatment, of, xxxix. 250; observations respecting the use of colchicum, in, 400; observations on the composition of Wilson's specific for the cure of, xl. 146; observations on the use of colchicum, in the cure of, 396.

GRAINGER, Mr. account of a ball found in the heart of a buck, xxxvi. 426.

GRANVILLE, Dr. case, in which the uterus was found after death to be imperfect on one side, xxxvi. 426.

GRATTAN, Dr. observations on the epidemic fevers of Dublin, xl. 305, 304.

GRAVEL, review of M. Magendie's Treatise on, xl. 339.—*See* CALCULI.

GREEN, Dr. observations on the use of oil of turpentine, in some cases of abdominal obstruction, xxxvi. 359.

GRIMME, Dr. account of an opening into the uterus through the vagina, for the extraction of the fœtus, xxxvii. 365.

GUIANA, account of the state of medicine in, xxxvi. 341.

GUADALOUPE, account of some skeletons found at, xxxv. 244.

GRIFFITH, Dr. review of his treatise on diseases of the liver, xxxvii. 143.

GUTHRIE, Mr. case of a wound of the peroneal artery, xxxvii. 500; observations on venereal diseases, xxxix. 236.

H.

HALE, Dr. observations on animal heat, xxxv. 291.

HALL, Dr. Marshall, account of some deletereous effects of tobacco, xxxv. 151; contribution to diagnosis, xxxvii. 240.

———, Dr. John, observations on fever, xxxvi. 368.

HALLIDAY, Dr. remarks on the state of lunatic asylums, xxxvii. 409.

HAMILTON, Mr. W. account of the rise, progress, and treatment, of a fever, in the neighbourhood of Ipswich, xxxvii. 451.

————, Dr. observations on the origin of syphilis, xl. 546.

HARLE, Dr. observations on epilepsy arising from disease of the spinal marrow, xxxvii. 262.

HARRISON, Mr. J. S. account of a new test for arsenic, xxxviii. 252.

————, Mr. T. observations on small-pox after vaccination, xxxvii. 2.

HARTLEY, Dr. remarks on some part of his psychological doctrine, xxxix. 96.

HATCHETT, Mr. observations on the mode of remedying damaged corn, xxxvii. 156.

HAVILAND, Dr. account of the fever which prevailed at Cambridge in 1815, xxxviii. 484.

HAYES, Dr. account of a case of spina bifida, xxxix. 183.

HEAD, observations on the formation of, in its relation to the intellectual faculties, xxxv. 52; case of uncommon disease of the, xxxvii. 18; remarks on that case, 272; cases illustrative of the ill consequences of blows on the head, 49, 50; case of extraordinary gun-shot wound of the, xxxix. 102; observations on the use of belladonna, in some painful affections of the, 141.

HEART, experiments and observations on the influence of the nervous system on the action of the, xxxv. 120; observations on the functions of the, 127; case of malformation of the, 147; three cases of inflammation of the, 223; observations on the causes of the motion of the heart, 260; observations on the connexion of its action with the influence of the brain, 317; case of remarkable disease of the, xxxvi. 51; observations on rheumatic affections of the, 200; observations respecting the force of the, xxxvii. 55; on inflammation of the, 323; case of enlargement of the, 416; observations on rheumatism of the, 466; case of very remarkable aneurism of the, xxxviii. 186; observations on the active dilatation of the, 257; account of a singular conformation of the right auricle of the, 342; case of gun-shot wound of the, xxxix. 404; observations on several organic diseases of the, 418; case of ossification of the, 87; case of palpitation of the, 9; observations on disease of the, connected with fever, 61, 62; on the effecting of rheumatic inflammation on the, 252; case of rupture of the, xl. 425; observations on aneurisms of the, 443, 444; remarks on the agency of the nervous system on the action of the, 437; case, in which there existed a communication between the ventricles of the, by an opening in the septum, xl. 9.

HEAT, observations on the efficacy of high degrees of external, in some diseases, xxxv. 170; observations on the use of, as an application in gout and rheumatism, xxxviii. 284.

———, *animal*, observations on the causes of its production, xxxv. 291; observations on its dependance on nervous influence, 332; observations on the influence of the nervous system, in regulating, xxxvi. 486; observations on the manner of its production, xxxvii. 283; on the evolution of, during the coagulation of the blood, 387; Mr. John Hunter's opinions respecting, xxxix. 254.

HEATH, Mr. observations on the generation of the guinea-worm, xxxv. 335.

HEBERDEN, Dr. extract from his observations on diseases of the prostate gland, xxxix. 314.

HEDGE-HOG, description of the, xxxvii. 431.

HELLEBORE, *black*, account of the poisonous effects of the, xxxix. 481; observations on its use in amenorrhœa, xl. 16.

HELLING, Dr. history of a case of ophthalmia fungosa, xxxviii. 179.

HÆMORRHAGE, observations on the use of the ligature as a means of preventing, after operation, xxxv. 190; observations on spontaneous, from various parts, 450; observations on the treatment of that from the uterus, xxxvi. 489, 514; observations on family disposition to, xxxvii. 174; observations on uterine, 532; observations on uterine, after delivery, not evacuated by the vagina, xxxviii. 462; case of fatal, from the extraction of a tooth, xxxix. 76; account of a family disposition to, xl. 429.

HENBANE, observations on its use, in inflammation, xxxvii. 163.

HENDRIKZS, Dr. description of some surgical operations, xxxviii. 420.

HENNEN, Mr. observations on venereal diseases, and on their cure without mercury, xxxix. 412; review of his work on Military Surgery, xl. 155, 214.

HENNING, Dr. observations on scarlet fever, xxxvii. 177.

HENRY, Mr. Louis, account of a bony substance extracted from the rectum of a man, xxxv. 108.

HEREDITARY DISEASES, some observations on, xxxvi. 501.

HERNIA, observations on hernia congenita, xxxv. 109; case of, of the stomach, through a passage in the diaphragm, xxxvi. 232; history of a case of strangulated, attended by some extraordinary circumstances, 453; case of, of the dura mater, xxxvii. 510; statistical accounts, respecting, 252; cases of strangulated, followed by an anus at the groin, xxxviii. 476; cases of hernia cerebri, xxxix. 71, 144; account of a case of inguinal, where the spermatic cord passed before the sac, xl. 151; reference to similar cases, related by Blizard, Schmucker, and Le Dran, ibid.; remarks on that, where the viscus protrudes, within the tunica vaginalis of the cord, but covered by another elongation of the peritoneum, 151, 152; observations on the state of the peritoneal sac, in, 224; remarks on crural, 153; on the division of Gimbernat's ligament, in the operation for strangulated, ibid.; case of umbilical, accompanied with stricture and rupture of the colon, 13; case of congenital, with a peritoneal sac enveloping the protruded intestine, 265; case of strangulated inguinal, terminating in sphacelus, cured without the occurrence of permanent artificial anus, 543.

HERODICUS, account of his gymnastic medical measures, xxxvii. 246.

HEY, Mr. jun. observations on his father's claim to the improvement in the operation of amputating the foot, xxxvi. 448; observations relative to parturition, xxxvii. 91; observations on the influence of the venereal disease on the fœtus, 335.

HICCOUGH, history of a severe case of, cured by colchicum, xxxvi 444.

HILL, Mr. George Nesse, review of his treatise on Madness, xxxv. 488; history of a fatal case of intestinal disease, xxxvi. 250.

HOARE, Mr. observations on vaccination, xxxvi. 9; account of some curiosities in natural history, xxxviii. 35.

HOLLAND, Dr. account of the pellagra of Italy, xxxix. 24.

HOME, Sir Everard, observations on the structure of the feet of the gecko, xxxv. 333; on the mode of action of specific medicines, 422; on the feet of flies, xxxvi. 253; on the fat of frogs, 165; on the origin of fat, xxxvii. 507; analysis of fossil bones, 522; on the passage of the ovum into the uterus, xxxviii. 76; xxxix. 395; on the medicinal qualities of colchicum, ibid.; xxxix. 400; on the ova of the sepia, and the vermes testacea, ibid.; account of the nests of the Java swallow, 401; on the glands of the human stomach, 402.

———, Dr. observations on his practice of inoculating with measles, xxxvi. 13.

HOOPING-COUGH, observations on its nature and treatment, xxxvi. 373, 438, 220, 383, 385, 438; xl. 26, 27, 28, 29, 30, 31.

HORNBAUM, Dr. on the poisonous effects of arsenic, xxxv. 338; on a species of ophthalmia peculiar to children, xxxviii. 185.

HOSACK, Dr. remarks on fever, xxxv. 355.

HOSPITALS, observations on the gangrene of, xxxv. 20; observations on the police of, relative to students, 421; observations on the purification of the air in, xxxvii. 167; an account of the surgical operations performed at that at Groningen, from 1810 to 1815, xxxviii. 420; observations on the gangrene of, xxxix. 14; xl. 216, 219, 226, 232.

HOTTENTOT, account of some peculiarities in the formation of the female, xxxvi. 466.

HOUSE-LEEK, observations on its medicinal qualities, xxxvi. 521.

HOWISON, Dr. case of morbus cœrulus, arising from ulceration of the lungs, xxxviii. 157.

HOWSHIP, Mr. observations on the formation of bone, xxxvi. 157; xxxvii. 325; xxxviii. 48, 195.

HUFELAND, Professor, observations on the use of henbane, in inflammation, xxxvii. 164; history of a case of disunion of a recently-united fracture of the humerus, xl. 538.

HUME, Mr. observations on the tests for arsenic, xxxv. 513; xxxvi. 239; xxxix. 27, 466; xl. 281, 407.

HUMERUS, histories of a peculiar affection of the, xxxviii. 355; xl. 364.

HUMPHRIES, Mr. observations on the fever prevalent at Gibraltar, xxxv. 505.

HUMULUS LUPULUS, observations on its medical qualities, xxxvi. 456.

HUNT, Mr. history of a case of congenital hernia, xl. 265.

HUNTER, Mr. John, his discoveries respecting the descent of the testicle, xxxv. 109; his observations respecting death, 126, 317; his observations on diseases of the prostate gland, 219; his observations on the quantity of blood expelled from the heart at each systole, 262, 264; remarks on his opinions respecting the action of the heart, 317; his observations on adhesive inflammation, xxxvi. 316; some observations on venereal diseases, 333; remarks on his operation for aneurism, xxxvii. 504; his observations on the adhesion of divided parts, 515; some remarks on the language of, xxxviii. 28; his notions respecting life, 29; some account of the life of, 107; remarks on his physiological principles, xxxix. 2; eulogium on, at the National Institute of France, 85; his observations on tumefaction of bones, 149; remarks on his doctrine of the vitality of the blood, 254; his remarks on microscopic evidence, 397; Hunterian oration at the Royal College of Surgeons, in 1818, 253.

————, Dr. William, remarks on his claims to the origin of the doctrine of the secretion of pus, xxxix. 285.

HUTCHISON, Mr. A. C. practical observations on surgery, xxxviii. 62; account of a case of nasal operation, xl. 322.

HYDATIDS, observations respecting those existing within the cranium in sheep, xxxvi. 428; account of one having the power of voluntary motion, taken from the brain, xxxviii. 257.

HYDROCELE, observations on the treatment of, xxxv. 220.

HYDROCEPHALUS, observations on the origin of, especially from disorder of the gastric system, xxxv. 88; observations on its origin, from inflammation of the membranes of the brain, 340; case of, terminating unsuccessfully, under the use of calomel and digitalis, xxxvi. 100; case of, unattended with the usual symptoms, with remarks, 388; observations on, xxxvii. 93; remarks on an extraordinary case of chronic, 344; observations on the frequency of acute, 351; case of, in a sheep, 431; case of hernia of the dura mater, connected with, 510; case of, followed by rupture of the brain and its membranes, xxxviii 464; case of, with dropsy of the periosteum, xxxix. 197; observations respecting its connexion with intestinal disorder, 95; remarks on the increase of, 470; observations on its effects, in the fœtus, xl. 431; case of, with observations, 306; observations on the use of moxa, in the cure of, 529.

HYDROGEN GAS, observations on its influence, in the production of acidity, xl. 99; on its effects on the oxide of silver, 168.

HYDROPHOBIA, case of spontaneous, xxxv. 14; case of, which terminated fatally, under the use of copious bleeding, calomel, and musk, xxxvi. 77; another case, of a similar nature, 79; observations on the treatment of, 479; account of the appearances on dissection, in two cases of, 517; on the use of the meloe proscarabæus in the treatment of, 519; history of a case of, xxxviii. 258; observations on the use of the alisma plantago, in the treatment of, 435; account of the use of the alisma plantago, for the prevention of, xl. 76, 453; account of the Russian practice of giving the blood of the rabid animal, as a preventive of, 119.

HYGIENE, general observations on, xxxvi. 218, 219.

HYPOCHONDRIASIS, observations on the causes and treatment of, xxxvi. 392; xxxvii. 243; xxxix. 349.

HYSLOP, Mr. account of a case of incontinence of urine, cured by bougies, xxxv. 411.

I.

IMPREGNATION, remarks on the process of, xxxv. 416.

INDIGOGENE, account of its discovery and chemical properties, xl. 115.

INFLAMMATION, observations on its nature, expressly considered to depend on paralysis of the muscular fibres of the arteries, xxxv. 13; observations on its nature, and the means of suppressing it, 105; remarks on the obscurities of its symptoms, in affections of the brain, 295; on the theory of, applied to fever, 354; observations on its nature and effects, xxxvi. 240; observations on the use of cataplasms, in internal, 251; observations on the adhesive effects of, 421; observations on the adhesive process of, xxxvii. 515; observations on the efficacy of henbane, in the cure of, 163; observations on the process of adhesive, xxxviii. 11; observations on the use of cold, as a remedy for, 103; remarks on some hypotheses of, 163; observations, illustrated by a case, respecting the adhesive consequences of, 250, 251; observations on some of the causes of, 374; observations on the recurrence of, during convalescence from fever, xxxix. 451; remarks on the suppurative form, without ulceration, 286; remarks on inflammatory diseases in general, 373; some observations on the varieties of its character, as it affects the brain, xl. 315.

INGENHOUSZ, Dr. biographical account of, xxxviii. 377.

INSECTS, some observations on the natural history of, xxxvi. 209, 253.

INSOLATION, observations on its effects, in some diseases, xxxv. 156.

INSTRUMENTS, *surgical*, description of some improvements in the form of several, xxxv. 199, 396, 443; xxxvii. 265; xl. 324.

INTESTINES, case of death, from obstruction of the, by a cocoa-nut, xxxv. 100; account of the removal of a piece of bone from the rectum, 108; on torpor of the, in affections of the brain, 305; remarks on the state of, in some diseases of children, 340; observations on the use of opium, in some diseases of the, 349; observations respecting the influence of a very cold climate, on the, 399; case of inflammation of the, with preternatural formation of the ilium, 436; case of obstruction of the, from a large biliary calculus, xxxvi. 69; case of mortification of the appendix coeci vermiformis, from the presence of a human tooth, 249; observations on the formation of fat in the, 303; observations on the efficacy of oil of turpentine, in some cases of abdominal obstruction, 359; observations on inflammation of the, 418; case of severe inflammation of the, from constipation, xxxvii. 263; fatal case of inflammation of the, 445; some observations on the functions of the, especially of the duodenum, xxxviii. 26, 27; case of strangulation of the, by an appendix of the ilium, 169; case of obstinate constipation of the, 280; xxxix. 473; observations on the state of, in fever, 50, 51, 60, 61; case of stricture and rupture of the colon, xl. 15; some observations on their action in the digestion of the food, 537.

IPECACUANHA, observations on the salt of, xl. 114.

IRITIS, observations on the origin and treatment of, xl. 504.

IRON, experiments on the influence of water on, xl. 104.

IRRITABILITY, experiments to determine the causes of some of the phenomena attributed to this, by the doctrine of Haller, &c. xxxv. 120, 121, 122, 124, 125; observations on that of the heart and arteries, during fever, 355; observations and reflections on, xxxvi. 124; remarks on muscular, xxxvii. 255; general observations on, xxxviii. 6, 7; remarks respecting its distinctness from sensibility, 157; observations on its influence in the production of disease, xl. 249; some observations on the Hallerian doctrine of, 434.

ISCHURIA, observations on the use of tobacco-glysters in some cases of, xxxv. 408; observations on the treatment of, xxxix. 39, 454.

ITARD, Mr. account of his method of treating psellismus, xxxix. 375.

J.

JACKSON, Dr. Edward, account of a case of encysted tumor, connected with the kidneys, xxxv. 1.

——————, Dr. Robert, some remarks on his opinions respecting fever, xxxvi. 155, 200; xxxvii. 311; xxxviii. 16; review of his treatise on Fever, xxxix. 45, 111, 464.

JAMES, Mr. observations on diseases of the heart, xxxix. 418.

JAMESON, Professor, on the natural history of the diamond, xxxix. 432.

JANIN, Dr. observations on the treatment employed in a case of syphilis, xxxix. 477.

JARDINE, review of his work on the Improvement of Surgical Instruments, xl. 324.

JAVA SWALLOW, account of the stomach of the, xxxix. 401.

JAUZION, Dr. history of a case of nymphomania, xl. 441.

JOINTS, observations on ulceration of the cartilage of the, xxxvi. 229; observations on coxalgia and rachialgia, xxxvii. 158; case of anchylosis of the, consequent on scarlet fever, 277; on rheumatic affections of the, 469; observations on injuries of the, from gun-shot wounds, xl. 164; observations on the white swelling of the, 222; observations on preternatural, with a case cured by friction, 427.

JOHNSON, Dr. James Rawlins, history of the medicinal leech, xxxvi. 52.

———, Mr. James, review of his work on Atmospheric Influence, xxxix. 247; case of disease of the brain, xl. 447.

K.

KARPE, Dr. on the use of affusion with diluted sulphuric acid in typhus, xxxv. 73.

KENEDY, Mr. case of remarkable disease of the arm, xxxvii. 219.

KERATONYXIS, observations on the operation of, xxxix. 135. — *See* Eyes.

KERN, Dr. observations on fracture of the membrane of the tympanum, xxxv 253.

KERR, Mr. George, remarks on the arteries, xxxvi. 94; xxxvii. 99.

———, Mr. James, observations on the poisonous effects of arsenic, and on the means of detecting it, in the animal fluids, xxxviii. 92, xxxix. 189.

KIDD, Mr. account of the fever epidemic in Ireland, xxxix. 408.

KING, Mr. case of obstinate constipation, xxxviii. 280.

KINGLAKE, Dr. Robert, observations on bleeding in peripneumonia, xxxv. 105; observations on obstetric practice by men, 174; further observations on the same subject, 363; further observations on the same subject, xxxvi. 3, 228; on the diagnosis between chronic hepatitis and phthisis pulmonalis, xxxviii. 37; observations on the use of blistering and topical cold, in various diseases, 102; observations on the comparative advantages of the cooling and heating treatment of gouty and rheumatic inflammation, 283; observations on morbid excess of vascular distention, 373; observations on febrile origin, xxxix. 371; on depressed vital action, 457; observations on excessive blood-letting, in maniacal affections, xl. 368; on hepatic congestion, 463.

KLAPROTH, Professor, account of the death of, xxxvii. 343.

KLEIN, Dr. notice of his work on Surgical Operations, xxxvii. 156.

KNIGHT, Mr. T. A. some phytological observations, xxxvi. 166.

KNOWLES, Mr. E. L. observations on the use of opium, in a case of violent contraction of the uterus, xxxvi. 331.

———, Mr. B. remarks on M. Larrey's memoir on the re-production of the vitreous humour of the eye, xxxvii. 455.

KRAFT, Dr. observations on blueness of the nose, in typhus, xxxv. 156; xxxvii. 256.

KREITSCHAAL, Dr. observations on the use of gas-baths, in some diseases, xxxvii. 527.

L.

LALLEMAND, Dr. review of his Thesis, xl. 343.

LAMBE, Dr. W. review of his additional reports, on the effects of a peculiar regimen, in cases of cancer, scrofula, consumption, and other chronic diseases, xxxvii. 394.

LEMERCIER, M. account of a case of wound of the eye-brow, producing some remarkable symptoms, xxxvii. 280; history of a case of neuralgia of the facial nerve, xxxix. 282.

LANGSTAFF, Mr. description of a fœtus found in the fallopian tube, xxxvii. 510; cases of fungus hæmatodes, xxxix. 155; case of fœtus contained in the fallopian tube, 429.

LARREY, M. observations on gun-shot wounds of the chest, xxxvii. 29; history of a remarkable case of paralysis, 157; observations on caries of the spine, 158; on some cases of blindness, 170; on wounds of the cornea, 257; on rheumatism, 466; on caries of the joints, 469; case of a wound of the head, attended with some singular phenomena, xxxix. 102.

LARYNX, observations on inflammation of the, xxxv. 413; xxxvi. 61, 400, 421; xxxvii. 155; xxxviii. 53; xxxix. 101; xl. 335.

LAURO-CERASUS, observations on its medicinal qualities, xl. 395.

LAWRENCE, Mr. William, histories of two cases of elephantiasis, xxxv. 210; observations on bronchotomy, xxxvi. 61; on the ligature of arteries, 227.

LEACH, Dr. account of some species of insects, xxxvi. 209.

LEECHES, account of a mode of using them by cutting off the tail, xxxv. 275, 352; on the varieties of, and on their tendency to produce erysipelas, 302; observations on the use of, from an early English work on surgery, 386; account of a general treatise on them, xxxvi. 52; observations on their peculiar advantages as means for the abstraction of blood, in some cases, xxxvii. 12.

LE GALLOIS, M. some remarks on his physiological opinions, xxxv. 120.

LEGH, Mr. account of his journey through Egypt, xxxvii. 474.

LEGS, observations on the treatment of ulcers of the, xxxv. 418.

LEMAIRE, M. observations on a peculiar structure of the teeth, xxxvii. 89, 441.

LEPROSY, histories of, from various authors, xxxv. 311; xxxviii. 21, 22; xxxix. 207.

LESLIE, Dr. account of a case of bite by a viper, xxxix. 161; observations on tic douloureux, xl. 476.

———, Professor, observations on a mode of producing ice, xxxviii. 173.

LETTSOM, Dr. biographical accounts of, xxxv. 204; xxxvii. 46, 374, 384; xxxix. 117, 130.

LEY, Dr. account of a case of puerperal fever, xxxviii. 117, 337.

LEUCORRHŒA, observations on the treatment of, xxxvi. 512.

LICHSTENSTEIN, extract from his Travels in Africa, xxxvi. 138.

LIFE, account of Bichat's researches on, xxxv. 41; experiments relative to the importance of different parts of the nervous system to the existence of, 119; reflections on the properties of, xxxvi. 120; observations and remarks on the principles of, xxxviii. 7, 8, 9; observations on depressed vital action, xxxix. 457; observations on the nature of vitality in the fœtus, xl. 435; some observations on the mode in which vegetable poisons destroy it, 436; facts shewing that the brain and spinal marrow are not necessary for its continuance in the fœtus, 435; some observations respecting the functions of animal life, 439.

LIGATURES, observations on the use of, on arteries, after operations, for the prevention of hæmorrhage, with some historical researches on this subject, xxxv. 190; xxxvi. 227; xxxvii. 378, 521; observations respecting their use in aneurisms, xxxvii. 378; account of an improvement in, 521; observations on the effects of, on arteries, xxxviii. 13, 14; remarks on the agency of, on arteries, 68, 69; observations respecting their effects on veins, xl. 521, 523; account of the consequences of their application to the nerves of the arm, 215; historical observations respecting the use of, 214; observations on their use in varicose aneurism, 215.

LIGHT, observations on the laws of its polarization, xl. 96; on the magnetic powers of the violet rays, ibid.

LIGHTNING, observations respecting its effects on animal bodies, xxxvi. 169; xxxviii. 517; xxxix. 81.

LIME, account of its crystallization, xxxvi. 258; on the mode of preserving the solution of, xxxvii. 449.

LITHOTOMY, observations on, xxxvi. 408, 519; xxxix. 76, 468; xl. 225.

LITTLETON, Mr. observations on the causes which influence the pupil of the eye, xxxvi. 89, 265; observations respecting the pulse, xxxviii. 164; further observations on the pupil, 283; observations on some phenomena of vision, xxxix. 354; on the utility of large doses of opium in various diseases, 447; observations on pertussis, xl. 26; on some phenomena of vision, 178; on specific gravity, 259.

LIVER, observations on the connexion of inflammation of the, with dysentery, xxxv. 402; observations on the causes, treatment, and mode of prevention, of inflammation of the, xxxvii. 143; observations on the disorders of the, especially as relates to the use of mercury in the treatment of, 144; observations on the diagnosis between chronic inflammation of the liver and phthisis pulmonalis, xxxviii. 32, 190; remarks on the treatment of slow inflammation of the liver, especially on the benefit of small and repeated bleedings, calomel, laxatives, &c. 33, 34; observations respecting its state in fever, xxxix. 50, 51, 60, 61; observations on the state of the, after death, in two habitual drunkards, xl. 299, 300; case of abscess in the, 29, 306; observations on the influence of disease of the, in the production of hysteria, 23; observations on congestion in the, 403.

LIZARD, instances of two being found enclosed in blocks of coal, xxxvii. 162; xxxviii. 35.

LOBEL, Professor, on a new operation for the cataract, xxxvii. 216, 273.

LOBSTEIN, Professor, observations on the medicinal qualities of phosphorus, xxxvi. 337.

LONDON, mortality and births in, in the year 1815, xxxv. 162; observations on the medical topography of, xxxviii. 84; some observations respecting the epidemic fever of, in 1818, xxxix. 34, 167; mortality and births in, in 1817, 172.

LUCAS, Mr. case of pneumonia, xxxvii. 239.

LUNGS, observations relative to their agency in the circulation of the blood, xxxv. 55; observations on the use of bleeding, in inflammation of the, 105; observations on the influence of the functions of the, on the rest of the economy, 130; various observations on diseases of the, and on the influence of different degrees of temperature on them, 182; some observations on inflammation of the, and on hæmorrhage from the, 457; observations respecting inflammation of the, expressly on the obscurity of the symptoms in some cases, xxxvi. 234; case of inflammation of the, accompanied with bilious fever, xxxvii. 239; observations on blood-letting, in inflammation of the, 410; case of inflammation of the pareuchynatous structure of the, 55; case of the lodgment of a foreign body in the, xxxix. 522.

M.

M'AVOY, Miss, observations respecting her powers of touch, &c. xxxix. 84, 115, 163, 474.

MAC GRIGOR, Sir James, observations on phthisis pulmonalis, as it affected the army in Portugal, xxxvi. 214; extracts from his medical history of the British army, 233.

MACHELL, Mr. description of his inhaler and vapour-baths, xxxix. 89; of his enema apparatus, xl. 198.

MAC LEAN, Dr. case of hysteria accompanied with trismus, xl. 15; case of calculus found in a bitch, 288.

MADEIRA, observations on the climate of, xxxvii. 364.

MAD-HOUSES, reports on, xxxv. 28, 509; xxxvi. 33, 109; xxxvii. 109, 409, 413; observations on the arrangement of, xl. 190, 293.

MAGENDIE, M. on the use of prussic acid, in several diseases, xxxix. 81.

MAGNESIA, observations on its use, in diabetes, xxxix. 266.

MAHON, Dr. observations on amenorrhœa, xl. 381.

MAKETY, Mr. observations on ophthalmia, xxxvi. 415.

MALTA, some account of the plague at, xxxv. 152, 319.

MANGILI, Professor, observations on the venom of the viper, xxxvii. 434.

MANIA, account of a case of demonomania, xxxv. 74; observations on the connexion of certain species of, with certain forms of the skull, 72; observations on its ordinary causes, 268; case of, from inflammation of the meninges of the brain, 297; observations on the efficacy of dulcamara and belladonna, in the cure of, 337; observations on the nature and origin of, especially considering it as a corporeal and symptomatic disease, 488; its division into sthenic and asthenic, 490; some illustrations of the influence of excessive mental exertion, in the production of, 397; case of, arising from loss of sight, 399; general treatise on, xxxvii. 241; observations on its connexion with delirium, 243; remarks on its frequency in Scotland, 409; some remarks on Spurzheim's doctrine of the nature and causes of, xxxviii. 23, 24, 25; observations on the use of cold application to the head, in the cure of, 104; remarks on Spurzheim's doctrine of, xxxix. 4; some observations respecting its origin from fever, 52; observations on its analogy to sanity, xl. 121; remarks on the different disordered impulses of the will, in, 122; remarks on the appearance of the countenance, in, previously to its occurrence, 124; on the sensibility to heat and cold, in, ibid.; remarks on the moral causes of, 123, 126; remarks on the means of ascertaining its existence, 188; on the use of restraint, in, ibid. 397; remarks on periodical exacerbation of, 189; on the appropriate places for the reception of the patients of, 190; remarks on the importance of attending to the spontaneous salutary changes of the system, in, 191; on the use of mercury, in, 192; observations on excessive blood-letting, in, 368; abstract of the doctrine of M. Pinel respecting, 479; observations on its connexion with displacement of the abdominal viscera, 543; on its origin, in puerperal women, 542.

MANIACS, report of the Committee of the House of Commons on the state of the public receptacles for, xxxv. 28; further account of the same subject, xxxvi. 33.

MANNA, observations on that produced from the Calabrian ash, xxxix. 326.

MANOURY, Mr. observations on a wound of the testicles, xxxix. 344.

MANTELL, Mr. account of an hybernating hog, xxxv. 511; account of a fossil alcyonite, xxxvi. 212; case of enteritis, 445.

MARCET, Dr. observations respecting the tests for arsenic, xxxv. 337; on the nature of the chyle, xxxvi. 237; on tests for arsenic, 238; on the medicinal properties of stramonium, xxxvii. 337; review of his work on Calculous Disorders, xxxix. 313.

MARCUS, Dr. observations on hooping-cough, xxxvii. 220.

MARSHALL, Mr. observations on the lauruscinnamomum, xxxvii. 522; review of his treatise on Arsenic, xxxviii. 240; on the medicinal qualities of salt, xxxix. 368.

MASKELYNE, Dr. directions for observing fire-balls, xxxix. 341.

MAUNON, M. description of a monstrous fœtus, xxxvii. 151; observations on vision, 496.

MATERIA MEDICA, observations on the action of specifics, xxxv. 422; observations respecting the doses of the, xxxvi. 104; observations on some new articles of the, from marine plants, 182; some observations respecting the articles of that of the natives of Guiana, 341.

MEAD, Dr. extract from his Observations on Diseases of the Genital Organs, xxxix. 205.

MEASE, Dr. account of the death of Dr. Rush, xxxvii. 81, 384.

MEASLES, observations on the treatment of, xxxvi. 10; on inoculation with, 13

MEDICAL JURISPRUDENCE, account of the laws relating to inoculation with the small-pox, xxxv. 66, 155, 202; observations on, connected with chemistry, xxxvi. 429; observations on the laws relative to surgeons, 146; on the laws relating to apothecaries, 490; some observations on, 429.

MEDICAL STATISTICS, the London bills of mortality for 1815, xxxv. 162; bills of mortality for St. Petersburgh, in the year 1812, 163; observations respecting the ages in which the greatest mortality in children takes place, 237; on the proportion of the mortality in parturient women and new-born children, 286, 288; observations on the diseases especially prevalent amongst the poor, 328; on the mortality, in child-bed, xxxvi. 197, 199; some remarks on the prevalency of consumption in the British army in Spain and Portugal, 214; on the proportion of the number of monsters to that of well-made children, 219; bills of mortality for London, in 1817, xxxix. 172; account of the population of England, xl. 209.

MEDICAL TOPOGRAPHY, of New Orleans, xxxv. 398; observations on that of London, xxxviii. 84; of some parts of Italy, 345; of the islands of the Mediterranean Sea, 441; of the north-eastern part of Asia, xxxix. 253.

MEDICINE, some remarks on the modern study of, xxxvi. 191; observations on the study of, 508; retrospect of the progress of, from January to July 1817, xxxviii. 1; account of the state of, amongst the people of the Tonga Islands, 198; remarks on the system of Cullen, 155; historical sketch of the progress of, from July to December 1817, xxxix. 1; from January to July 1818, xl. 1; observations on zootic, 5; observations on the medical measures instinctively employed by brute animals, xl. 486.

MEDITERRANEAN, some observations respecting the medical topography of the, xxxvii. 465; xxxviii. 441.

MEEKER, Dr. case of preternatural joint cured by friction, xl. 304.

MELÆNA, histories of some cases of, xxxix. 495; cases of, removed by oil of turpentine, xl. 304.

MELOE, some observations respecting the history of, xxxvi. 519.

MENSERT, Dr. on the operation of keratonyxis, xxxix. 134.

MENSTRUATION, observations on, xxxv. 458; instances of it, at an advanced period of life, xxxvii. 261; observations on the efficacy of black hellebore, in restoring suppressed, xl. 16; observations on suppression of, 381.

MENORRHAGIA, observations on the treatment of, xxxv. 328.

MENURET, Dr. memoirs of, xxxvii. 206.

MERCURY, account of its presence, in the vessels of animals on whom it was employed by the way of inunction, xxxv. 74; observations on its use in the cure of the plague, 152; observations on its deleterious influence on the brain, 269; observations on the use of, in the treatment of fever, 407; case, in which a child was salivated by sucking a woman using mercury, 512; observations respecting its use in gout, xxxvi.

505; account of some remarkable effects of the oxy-muriate of, xxxvii. 157; observations on its use, in diseases of the liver, 143; remarks on its use, in yellow-fever, xxxviii. 487; observations respecting its use, in fever, xxxix. 57; account of a new mode of preparing the submuriate of, xl. 106.

MERRIMAN, Dr. some observations respecting the practice of midwifery, xxxv. 282.

METASTASIS, of disease, a frequent cause of mania, xxxv. 268; remarks on that of various functions, 456, 457, 458; observations respecting that of rheumatic inflammation, xxxvi. 201; remarks on that of rheumatic inflammation, xxxvii. 466.

METEORS, directions for observing them, xxxix. 341.

METEOROLOGICAL OBSERVATIONS, for London, xxxv. 78, 167, 255, 343, 431, 519; xxxvi. 87, 175, 263, 351, 437, 525; xxxviii. 87, 177, 265, 351, 439, 521; xxxix. 87, 175, 263, 351, 439, 527; xl. 86, 177, 262, 358, 456, 551.

MICROSCOPE, on the use of the, in anatomical observations, xxxix. 397.

MIDWIFERY, remarks on the practice of, by men, xxxv. 3; historical observations respecting the practice of, 84; observations on the practice of, by men, 174; observations on some of the consequences of the practice of, by women, 238; further remarks on the same subject, 282; further observations on the same subject, 348, 351, 359; xxxvi. 3, 96, 197, 288, 365; remarks on the importance of the profession of, xl. 17.

MILLER, Mr. case of palsy, xxxvi. 362.

MILLINGTON, Mr. observations on small-pox succeeding to vaccination, xxxv. 477.

MILLS, Dr. on the morbid anatomy of the brain in typhous fever, xxxviii. 491.

MIND, observations on its connexion with organization, xxxv. 46, 49; some general reflections on, and its influence on, and connexion with, the body, xxxvi. 129; observations on its influence on the body, 393; remarks on Spurzheim's doctrine of the nature of the faculties of the, xxxviii. 23, 24, 25; new analysis of the functions of the, xxxix. 96; some remarks on the influence of bodily derangement on the manifestation of the, 51, 52.

M'LOGHLIN, Dr. case of ovarian dropsy, xl. 306.

MOGOSTOKOS, on the necessity of accoucheurs, xxxvi. 197.

MONSTROSITY, statistical observations respecting the occurrence of, xxxvi. 219; account of a fœtus with two heads and two hearts, xxxviii. 338; history of a remarkable case of, xl. 84.

MONTAIN, M. history of a case of hydrocephalus, xxxvii. 344.

MONTEGRE, M. remarks on the formula of the ancient physicians, xxxvii. 342; remarks on his experiments on digestion, xxxix. 2.

MOORE, Mr. on an artificial pupil, xxxvi. 42; letter to, from the King of Hayti, 169; remarks on his history of vaccination, xxxix. 265.

———, Dr. Francis, observations on ophthalmia, xxxix. 109.

MORIL, Mr. case of gun-shot wound, xxxvii. 155.

MORGAN, Dr. remarks on his claim to the doctrine of the secretion of pus, xxxviii. 196; xxxix. 169, 285.

MORPHINE, experiments and observations respecting its effects on the animal economy, xl. 114, 275.

MORRISON, Dr. review of his treatise on Tetanus, xxxvii. 317.

MORVEAU, M. Guyton de, some account of his life and character, xxxvii. 167; xxxviii. 382.

MOXA, observations on its use, in caries of the spine, xxxvii. 158; in some diseases of the eyes, 170.

MURRAY, Mr. account of an epidemic affection of the throat, xxxvi. 47.

———, Dr. on definite proportions in chemistry, xxxix. 431.

MUSCLES, remarks on the discovery of the use of the oblique, of the eye, xxxvi. 251; on the influence of moisture on the irritability of the, xxxvii. 255.—*See* Muscular Motion.

MUSCÆ VOLITANTES, remarks on, xxxix. 103, 106; xl. 182.

MUSCULAR MOTIONS, experiments on the influence of the different parts of the nervous system on, xxxv. 120; observations on the nature of involuntary, 260; some general observations on, xxxvi. 124; observations on Dr. Mayow's claim to the discovery of the action of the oblique intercostal muscles, 251; some observations on the course of muscular fibres in the state of contraction, 254; observations respecting the influence of the ganglionic system of nerves, in the production of, xl. 438.

MUSHROOMS, proposal of milk, as a remedy for the poisonous effects of, xxxv.

72; on the danger of mistakes respecting, xxxvi. 451.

N.

NATURAL HISTORY, account of the torpedo, xxxv. 333; account of an extraordinary prevalence of caterpillars in Switzerland, 337; of the species of insects, *acarei*, 466; of the medicinal leech, 52; account of the sea long-worm, (*gordius marinus*, LIN.) 207; a tabular view of the external characters of four classes of animals which Linnæus arranged under *insecta*, &c. 209; description of a fossil alcyonium, 212; account of the mechanism by which insects walk contrary to gravity, 258; account of the structure of the genitals of the female Hottentot, 466; account of an adder found in a block of coal, xxxvii. 431; account of a lizard found in a bed of chalk, 162; account of some animals found alive, enclosed in masses of stone, &c. xxxviii. 35; account of the hedge-hog, 431; of the worms found in the human body, 434; observations respecting the hybernation of swallows, 519; some observations respecting the orang-outang, xxxix. 431; account of the mode of breeding of snails, 400; some observations respecting the influence of climate on the temperature of animals, xl. 452; account of a female dwarf, 81; historical account of meteoric stones, 137; on the influence of music on the mouse, 257; account of the milk of the cow-tree, 258; observations on specific gravity, 269; on water-spouts, 78; account of a change of colour of the skin from black to white, 391; account of the proteus anguinus, 452.

NAVY, some remarks on the practice of medicine in the, xxxv. 499; xxxvi. 234, 335; xxxviii. 411, 441.

NEGROES, account of the earth-eating, xxxviii. 79.

NERVOUS SYSTEM, on the division of, into those of animal and organic life, xxxv. 42; on the influence of each of those systems, 52; observations and experiments on its relations to the sanguiferous, 119; on its influence on the action of the heart, 261; remarks on its influence on the action of the heart, 317; account of some experiments to determine its influence in secretion, 331; observations respecting the identity of the influence of the, and galvanism, 423; observations on the use of galvanism, in some affections of the, xxxvi. 165; and on the identity of galvanism and the nervous fluids, 165; observations on various mordid affections of the, 392; observations on its influence in regulating the heat of the body, 486; observations on hypochondriasis, xxxvii. 243; on the efficacy of colchicum in that affection, 17; general observations on the functions of the different parts of the, 132; review of a general treatise on the relations of the, xxxviii. 495; observations on the influence of irritation of the, in the production of disease, xl. 249; observations respecting the influence of ligatures on the nerves of the arm, 215; general remarks on its functions, 346; comparison of those of man with that of brutes, 440; the opinions of Reil, Prochaska, Scarpa, and Legallois, respecting the production of the power of the, in the nerves themselves, 438; some axioms respecting its influence, 49; observations on that part of it necessary to organic life, 346; observations on injuries of the nerves, by gun-shot, 215.

NEURALGIA, observations on, especially on the use of belladonna, in some cases of, xxxix. 141; case of, of the facial nerve, 282; observations on the use of the actual cautery, in some cases of, 257; case of severe, affecting the thumb, 77; various observations on, 176, 283, 519.

NEWNHAM, Mr. review of his work on retroversion of the Uterus, xl. 325.

NEW ORLEANS, medical topography of, xxxv. 378.

NICHOLL, Dr. account of a case of imperfect vision, xxxvii. 331.

NICHOLLES, Mr. J. on the structure of the teeth, xxxvii. 441.

NITRATE OF SILVER, some observations respecting its effects on the animal body, xxxv. 76, 132, 337; observations on its use, in several diseases, 453.

NITRE, account of its effects on animal bodies, xxxix. 160.

NOMENCLATURE, *medical*, remarks on, xxxv. 143; xxxviii. 274.—*See* NOSOLOGY.

NOSE, observations on the blue colour of, in typhus, xxxv. 298; xxxvii. 256; on the restoration of a lost, xxxvi. 169.

NOSOLOGY, review of Mr. Good's system of, xxxviii. 210, 306; various remarks on, xxxv. 414; xxxvi. 117, 156, 158, 327, 449; xxxix. 461; xl. 183.

NYMPHOMANIA, history of a case of, xl. 441.

### O.

OBESITY, observations on, xxxvii. 245, 262, 528.

O'BRIEN, Dr. observations on the fevers of Dublin, xl. 398; case of exterior suppuration of the liver, 288.

ODIER, Professor, account of the illness and death of Professor Saussure, xxxvii. 70.

OLIVE, observations on the culture of the, xxxix. 519.

OPERATIONS, *surgical*, account of Chopart's mode of partial amputation of the foot, xxxv. 74; remarks on that of sounding the bladder, 81; on the puncture of the membrane of the tympanum, 253; observations on the revival of the talliacotian, 293; account of an instance of that of tying the iliac artery, 363; account of Assalini's improved instruments for, 396; account of the successful application of a ligature to the trunk of the carotid artery, 411; case of the application of a ligature to the subclavian artery, 412; case of the successful performance of bronchotomy, 417; observations respecting excision of the uterus, 426; for forming an artificial pupil, xxxvi. 49; on those performed on the trachea, 61; amputation at the shoulder-joint, 108; observations on the ligature of arteries, 227, 235; observations on the extraction of the placenta, 286; on the excision of the uterus, 295; on amputation at the instep, 355, 448; on the extraction of the placenta, 363; observations on that for the stone, with remarks on the different modes of effecting it, and on the different instruments, 408; on that performed for hydatids within the cranium, in sheep, 428; case of trepanning, followed by hernia cerebri, 449; account of the first, for the stone, 519; at the instep, xxxvii. 354, 522; improved, for aneurism, 377, 500; case of ligature of the iliac artery, 509; account of various, for the cataract, 211, 216, 273, 455, 496; reference to a view of the results of various, 156; account of that, for varicose veins of the legs, 155; some remarks on the use of the tourniquet, xxxviii. 63; account of some new instruments for performing those necessary in some cases of parturition, 91; for the removal of a large tumor from the orbit of the eye, 179; case of ligature of the internal iliac artery, with an account of the origin of that operation, 267; account of those performed in the Academical Hospital at Groningen, for 1810 to 1815, 420; observations on the different modes of amputating at the foot, xxxix. 523; observations on the Cæsarian, 13; observations respecting the talliacotian, 16; case of laryngotomy, 101; some historical observations respecting lithotomy, 76; case of, 408; observations on the appropriate period for amputation, in cases of gun-shot wounds of the extremities, xl. 159, 221; ligature of the femoral artery, for aneurism of the femoral vein, 215; observations on lithotomy, 225; account of the excision of a portion of the ribs and plura, by M. Richeraud, 272; case of that for forming an artificial nose, 322; account of extirpation of the uterus, 325; removal of a tumor of os humeri at the joint, 221, 364; removal of a large tumor from the angle of the jaw, 62; case of ligature of the aorta, 511; cases of ligature of the brachial and carotid arteries, 519; account of the removal of a calculus from the bladder, by a new mode of extracting it through the urethra, 545; some remarks on those for strangulated hernia, 151, 153, 224, 543.

OPHTHALMIA, observations respecting the contagious nature of some forms of, xxxvi. 415; observations on, connected with an affection of the head, xxxvii. 14; observations on a species of, especially affecting sickly children, xxxviii. 185; observations on the Egyptian, xxxix. 133; remarks on Sir William Adams's treatment of, in the army, 308; remarks on a form of, as connected with fever, 111.

OPIUM, observations on its efficacy, in colic, xxxv. 349; observations respecting the utility of, in diabetes, xxxvi. 26; observations respecting its efficacy, in violent contraction of the uterus during pregnancy, 331; observations respecting its efficacy in uterine hæmorrhage, 497; observations on the mode of administering it, xxxvii. 67; remarks on the impropriety of its use, in hooping-cough, 231; its efficacy, in dysentery, 314; remarks on the benefit of large doses of, in tetanus, 322, 371; observations on its efficacy in the venereal disease, xxxix. 207; remarks on the utility of large doses of, in many diseases, 447; account of some experiments made with the salt of, xl. 275; experiments and observations on the effects of the salts of, on the animal economy, 84, 275.

ORANG-OUTANG, anatomical description of the, xxxix. 431.

OSBECK, Dr. account of his mode of treating syphilis, xl. 340.

OSSIANDER, Professor, account of his mode of treating cancer of the uterus, xxxv. 426; xxxvi. 250, 293, 295.

OSSIFICATION, some observations on, xxxvii. 153, 209.—*See* BONES.

OTAHEITE, on the introduction of the venereal disease into, xxxix. 229.

OVUM, on its passage into the uterus, xxxix. 395.

OXYGEN GAS, observations on its combinations with chlorine, xl. 98; on its efficacy when respired, in alleviating the paroxysm of angina pectoris, 303.

OXYGEN, account of the late discoveries respecting it, xxxv. 115.

P.

PARALYSIS, observations on the efficacy of titillation, in the cure of, xxxv. 73; on that of the limbs of children, 242; of the heart, considered as the cause of the dilatation of the ventricles, 263; observations on the utility of stramonium, belladonna, and gratiola, in the cure of, 338; case of, from intoxication, xxxvi. 194; case of recovery from a severe case of, 362; observations on the efficacy of nux vomica, in the cure of, xxxvii. 157, 345; observations on the shaking species of, xxxviii. 71; observations respecting its origin from solution of continuity of the brain, xl. 351.

PARAPHYMOSIS, observations on its origin and treatment, xxxix. 205.

PARKINSON, Dr. account of his system of nosology, xxxvi. 327; outlines of a new system of nosology, xxxviii. 449; analysis of the human mind, xxxix. 97; nosological observations, 460; xl. 183; observations on scarlet fever, 477; case of intermittent fever, accompanied with inflammation of the brain, 476.

——————, Mr. James, review of his Treatise on the Shaking Palsy, xxxviii. 71.

PARKMAN, Dr. observations on insanity, xl. 120.

PARRY, Dr. review of his treatise on the Arterial Pulse, xxxv. 478; observations on the powers of the arteries, xxxvi. 277; review of his Elements of Pathology and Therapeutics, xxxvii. 51, 127.

PARTURITION, observations on the necessity of artificial aid during, and on the most severe accidents attending it, xxxv. 3, 174, 238, 283, 348, 350, 360; observations on the presentation of the placenta, expressly, 361; on uterine hæmorrhage, 362; further remarks on the necessity and propriety of artificial aid, in the act of, by Dr. Kinglake, 363; case of rupture of the uterus, terminating favourably, 449; case of presentation of the placenta, and observations on its consequences, 462; observations on the danger of, and on the propriety of artificial interference, in, on general and on particular occasions, xxxvi. 3, 4, 5, 6, 7, 8, 96, 197; observations on the best mode of extracting the placenta, 286; further observations on the difficulty and danger of, 288; observations on the mode of extracting the placenta, 363; observations on the use of ergot rye, in facilitating, 379; observations on the nature and treatment of uterine hæmorrhage, during, 491; observations on hæmorrhage, during, 514; observations on some remarkable cases of, xxxvii. 144; account of, amonst the Arabs, 257; case of premature, 15; observations on difficult cases of, 91; account of an unfortunate case of, 219; case of, effected by an incision through the vagina, 365; case of, effected by the Cæsarian operation, 194; case of premature, artificially excited, 15; observations on difficult, 91; cautions to be observed after, 220; account of some new instruments for aid in, xxxviii. 91; observations on internal hæmorrhage, after, 462; observations on the Cæsarian operation, xxxix. 13; observations on turning the fœtus, 159; observations on the use of olive-oil, to facilitate, 80; observations on phlegmasia dolens, consequent on, 12; case of placental presentation, with hæmorrhage, 443; observations on the occurrence of mania as a consequence of, xl. 542; case of death, from lingering, 19; reports of the results of the cases occurring in the Dublin Lying-in Hospital, 401; observations on uterine hæmorrhage during, 123, 402; on convulsions during, 403; observations on the treatment of laceration of the urethra and bladder, during, ibid.; on the management of cases where the funis presents, ibid.; observations on the practical indications furnished by the communication between the vessels of twins, 345; on retention of the placenta, 401, 403.

PASSIONS, physiological observations on the, xxxv. 50; observations on their influence on the functions in general, 129.

PATHOLOGY, (general observations on,) on the influence of gastric disorders, &c. on the functions of the brain, xxxv. 91; remarks on the obscurity attending that of the brain, 295; observations on the humoral, 353; various observations on, expressly relating to spontaneous hæmorrhage, 450; various observations and remarks relative to, xxxvii. 170; review of a treatise on, 519; review of Dr. Parry's Elements of, 51, 127; miscellaneous observations relative to, xxxviii. 48; some general reflections on, xl. 343.

PEARSON, Dr. G. some remarks on the London Pharmacopœia, xxxv. 345; observations on some diseases of the heart, xxxvi. 52; case of aneurism of the aorta, xxxvii. 420; case of hydrothorax, xxxix. 405.

————, Mr. J. case of neuralgia affecting the thumb, xxxix. 77.

PELLAGRA, description of the, xxxix. 211, 217.

PERCIVAL, Dr. E. description of an epidemic petechial febricula, xxxix. 493; on the medicinal qualities of green tea, 495; on dropsy, 499; account of some states of the abdominal viscera, in mania, and observations on the efficacy of turpentine, in maniacal epilepsy, 488; observations on the epidemic fever of Dublin, xl. 307.

PERAY, M. experiments on the adhesion of separated portions of animal bodies, xxxvi. 169; observations on wounds of the chest, xxxviii. 370; observations on ingurgitation, xl. 338.

PERIOSTITIS, observations on the origin and treatment of, xl. 64.

PERITONITIS, cases of, with remarks, xxxvi. 218; xxxviii. 105, 447; xxxix. 513.

PETERS, Dr. on the medicinal qualities of house-leek, xxxvi. 521.

PETTIGREW, Mr. extracts from his Life of Dr. Lettsom, xxxv. 204; xxxviii. 339, 374.

PHARMACY, review of a general treatise on, xxxv. 141; observations on the appropriate language for, 275; observations on the antimonial powder of the London Pharmacopœia, 345; on the preparation of the fucus vesiculosus, xxxvi. 186; account of the proper mode of preparing phosphorus for medicinal use, 337; observations on the drop measure for fluids, 426, 516; account of several new preparations in, xxxvii. 83; table for ascertaining the properties of several substances in, 363; account of some formula of the ancient physicians, and remarks on their mystical character, 342.

PHARMACOPŒIA, remarks on the London, xxxv. 141.

PHARYNX, case of ulceration of the, xxxvi. 403.

PHILADELPHIA, some statistical observations on, xxxvii. 403.

PHILIP, Dr. A. P. Wilson, account of his experiments on the relation subsisting between the nervous and sanguiferous systems, xxxv. 119; some remarks on that account, 316; experiments on the nervous influence on secretion, 331; on the identity of the nervous and galvanic influence, 423; remarks on a review of his paper, 460; reply, xxxvi. 46; observations on nervous diseases, 165; observations on a species of pulmonary consumption, xxxvii. 331.

PHILLIPS, Dr. E. accounts of a case of locked-jaw, xxxv. 408; account of some parts of a fœtus found in the body of a child, 411.

PHLEGMASIA DOLENS, case of, succeeding puerperal fever, xxxv. 14; observations on, xxxix. 12; case of, in a male, xl. 426.

PHOSPHORUS, observations on the medicinal qualities of, xxxv. 112; xxxvi. 337; observations on its combination with oxygen, xl. 102.

PHOSPHURETS, experiments with the, xl. 102.

PHTHIRIASIS, description of, xxxviii. 350.

PHYSIOLOGY, review of Bichat's Researches on Life and Death, xxxv. 41; Dr. A. P. Wilson Phillip's experiments on the relations subsisting between the nervous and sanguiferous systems, 119; notice of an elementary treatise on, 508; some remarks on M. M. Magendie's and M. Coutanceau's researches in, xxxvi. 81; account of Dr. Friedlander's Treatise on the Physical Education of Man, 218; observations on the nature of the process of organic formation, 240; various observations and remarks respecting, especially as relates to its dependance on chemical phenomena, 340; various observations on, 413; various observations and reflections on, xxxvii. 52, 127; general observations on, especially the modern theories, xxxviii. 6, 7; various observations and reflections on the functions of the nervous systems, xl. 215, 346, 438, 440, 44, 346, 435, 436, 439.

PIERSON, Dr. observations on the decarbonizing functions of the lungs and other organs, xl. 331.

PINEL, M. sketch of his doctrine of insanity, xl. 479.

PLAGUE, observations on that prevalent at Malta, xxxv. 151; another account of the plague at Malta, 319; account of, at Cairo, xxxvii. 477; observations on it, in Egypt, in general, 478; account of it, at Cyprus, 481; observations on, especially respecting its origin from contagion, xxxix. 491; some observations respecting its propagation at Morocco, xl. 292.

PLANTS, M. Humboldt's account of the species known to the ancients, and at the present time, 512; observations relative to the time of flowering of certain, xxxvi. 84; some observations on the physiology of, 166; observations respecting their time of flowering, 171; some observations on the medicinal qualities of marine, 183; observations on the season of flowering, &c. of certain, 258, 348; observations on the nature and causes of the ergot in rye, 376; account of those growing round London, xxxvii. 425, 533; xxxviii. 85, 349, 437.

PLATINA, various experiments and observations on its chemical compounds, xl. 106.

PLETHORA, general observations on its nature and consequences, xxxviii. 373.

POISONS, case of, by tobacco, xxxv. 151; account of the effects of the upas antiar, 330; on the means of determining the effects of arsenic, 338; observations on the deleterious agency of the spurious angustura, xxxvi. 160; account of the effects of the white briony root, 435; history of the deleterious effects of the agaricus campanulatus, 451; on that of oxalic acid, 517; experiments on the effects of that of the viper, 522; case of poisoning, by arsenic, xxxvii. 430; observations on the syphilitic, 36; remarks on the action of, 209; on the use of, by savages, 210; observations on poisoning with arsenic, xxxviii. 92, 95; observations on the poisonous effects of arsenic, 240, 252; case of death, from the bite of a viper, 517; observations respecting that of black hellebore, xxxix. 481; various observations on morbid, 13, 203, 271; remarks on poisoning by arsenic, 27; some observations respecting the mode in which some of the vegetable poisons destroy life, xl. 436.

POLYDYPSIA, cases of, with remarks, xxxv. 199, 201.

POLYPUS, case of, in the stomach, xxxvii. 433; case of, in the heart, 416.

POLYSARCHIA, remarkable case of, xxxvii. 262.

POOR, observations on some measures for affording medical assistance to the, xxxviii. 364, 511; xxxix. 34, 293, 297, 331, 332.

PORTAL, M. account of the illness of Madame de Stäel, xxxviii. 293; observations on vomiting, xxxix. 517.

POTEL, M. on the use of the actual cautery, in the cure of sciatica, xxxix. 257.

PORTUGAL, remarks on the diseases of the British army in, xxxvi. 214; on the treatment of the venereal disease in, xxxix. 228.

POST, Dr. cases of aneurism, xl. 531.

POWELL, Dr. observations on the pathology of the brain, xxxv. 295; on the regulation of mad-houses, 28.

PREDISPOSITION, remarks on the application of the term, xxxv. 495; xxxvi. 501; account of a family predisposition to hæmorrhage, xl. 429.

PREGNANCY, case of remarkable extra-uterine, xxxv. 273; case of, presenting some remarkable circumstances, 347; observations on the influence of the circulation of the mother on that of the fœtus, 134; case of rupture of the uterus during, terminating favourably, 440; observations on the efficacy of copious bleeding, and large doses of opium, in violent contraction of the uterus during, 331; cases of extra-uterine, xxxvii. 195, 510; case of extra-uterine, xxxix. 429; observations on the duration of, in different animals, 6; case of extra-uterine, xl. 343; observations respecting the development of the placenta, 344; observations respecting the formation of the deciduary membrane in the uterus, in cases of extra-uterine gestation, 344.

PREPUCE, observations on some diseases of the, xxxix. 306.

PRESCOTT, Mr. account of a case where death followed the swallowing of a chocolate-nut, xxxv. 100.

PRESSURE, observations on its efficacy, in a case of incontinence of urine, xxxv. 411; on its use, in the cure of ulcers of the legs, 419; on its use, in rheumatism, xxxvi. 318; its efficacy, in gout, 420; remarks on its use, in cancer, 461; xxxvii. 354; xxxviii. 478.

PREVOST, Mr. observations on the colours of bodies, xl. 256.

PRIDEAUX, Mr. on the use of nitrate of silver as a test for arsenic, xxxix. 297; xl. 467.

PRING, Mr. review of his work on the Nervous System, xxxviii. 495.

PRITCHARD, Dr. case of typhous fever, with remarks, xxxviii. 406.

PRIZE QUESTIONS, respecting rabies canina, xxxv. 70; on the motion of the sap in plants, 70; on the medical use of cold water, 72; xxxvi. 80; on syphilis and scrofula, 254; on the chemical changes effected on the air by the growth of plants, ibid.; respecting the pulse, 432; respecting fever, ibid.; on coction in diseases, ibid.; on the formation of milk, ibid.; on inspection of urine, 433; on factitious mineral waters, ibid.; on the contagious nature of phthisis, ibid.; on the treatment of phthisis, ibid.; on the diagnosis of the early state of phthisis, ibid.; on debility as a symptom in diseases, ibid.; on the properties of camphor, ibid.; on putrid fever, ibid.; on the diagnosis of measles, ibid.; on the contagious nature of hooping-cough, ibid.; on epilepsy, 434; on medical nomenclature, ibid.; on the topical use of opium in ophthalmy, ibid.; on the colostrum, ibid.; on the mode of exhibiting the super-oxide of mercury, the muriates of barytes, and of gold, ibid.; on the use of milk in cases of poisoning, ibid.; on the signs of the presence of worms in the intestines, ibid.; on the existing of muscular luxations, ibid.; on the existing of idiopathic fever, ibid.; on hydrophobia, xxxvii. 83; on a remarkable complaint, 275; on the changes produced on the air by the action of the skin, xxxviii. 79; on the utility of pathological anatomy, xl. 549; on cancer of the uterus, on the growth of the body, 549.

PROSTATE GLAND, some remarks on its diseases, xxxv. 218; xxxix. 314.

PROUST, M. analysis of barley before and after germination, xxxix. 297.

PROUT, Dr. observations on the urine, and its changes during disease, xxxix. 322; on the manna of the Calabrian ash, 326.

PRUSSIC ACID, observations on its medicinal qualities, xxxix. 81.

PSORA, observations on its origin, xxxv. 471.

PUERPERAL DISEASES, general observations on, xxxvi. 199.—*See* PARTURITION, *and* FEVER, *puerperal.*

PURDY, Dr. account of a case of phlegmasia dolens, in a male, xl. 426.

PURTON, Mr. observations on uterine hæmorrhage from placental presentation, xl. 138.

PULSE, *arterial*, observations on its frequency in different states, xxxv. 266; observations respecting the influence of disease of the brain, on the, especially as relates to its frequency, 297, 299, 300, 301, 302, 303, 307, 308, 310; experiments and observations on the nature of the, 479; on the indications it furnishes for bleeding, in fever, 502; observations on the, xxxvii. 100; observations respecting redoubling, xxxviii. 164.

PURGATIVES, remarks on those most appropriate for children, xxxv. 329; observations on the use of, in fever, 357; observations on the use of, combined with ipecacuanha, in the cure of dysentery, 400; account of those produced in Guiana, 342; observations on the abuse of, xxxviii. 38; observations on their uses in fever, 486; xl. 313, 400.

PURLITZ, Dr. case of mania, with remarks, xxxv. 338.

PURTON, Mr. on the identity of the nervous and electric fluid, xxxv. 104; on the extraction of the placenta, xxxvi. 363.

PUS, observations on the opinions entertained respecting its nature, xxxviii. 176; xxxix. 285, 286, 305, 353, 462.

PYM, Dr. observations on the Bulam fever, xxxv. 507.

PYROLA UMBELLATA, observations on its medicinal qualities, xl. 451.

### R.

RABIES CANINA, observations on the different forms of the, xxxv. 491; case of supposed, in a dog, removed by blood-letting, xxxvi. 71; observations showing the susceptibility of herbivorous animals to rabies, 337; observations on the use of the actual cautery, for the destruction of the poison of, 435; distinction between, and tetanus, xxxvii. 321.

RAMSAY, Dr. on the influence of oil and oiled skin applied to the human body, xxxv. 169.

RAVEN, Mr. observations on the use of colchicum, in chorea, xxxvi. 181, 292; case of obstinate hiccough, removed by colchicum, 444; on the efficacy of the same remedy in hysteria and hypochondriasis, xxxvii. 17.

READ, Mr. case of tetanus, relieved by opium, xxxvii. 372.
REDHEAD, Mr. cases of small-pox, after vaccination, xxxvii. 3, 8.
REEVES, Dr. on the influence of certain states of air and water on the system, xxxvii. 397.
REGIMEN, account of the influence of a certain form of, on various chronic diseases, xxxvii. 399.
REGNAULT, M. observations on the use of moxa, in hydrocephalus, xl. 529.
REHMANN, Dr. on the medicinal qualities of the ballota lanata, xxxvi. 346.
REID, Dr. John, of London, review of his Treatise on Hypochondriasis and Nervous Affections, xxxvi. 392.
REMER, Dr. on the influence of some atmospheric effluvia on the body, xxxvii. 167.
RENWICK, Dr. account of Miss M'Avoy, xxxviii. 291; xxxix. 114, 164, 174.
RESPIRATION, observations on the necessity of, to the functions of life in general, xxxv. 130; observations on the causes of, and its influence on the circulation of the blood, 264; observations on the mechanism of, xl. 31; observations respecting the action of the thoracic muscles, in the act of, 291; observations on the decarbonizing agency of the functions of, 331.
RHEUMATISM, various observations on the treatment of, xxxv. 168; instance of the efficacy of the warm bath in the cure of, 251; observations on rheumatic affection of the heart, xxxvi. 200; observations on the treatment of, by pressure, 318; on, as it affects the eyes, 382; review of a treatise on, 498; remarks on that, xxxvii. 199; observations on the benefits of bleeding, in, 314; observations on that affecting the heart, 324; on the use of ginger, in some cases of, 242; remarks on the metastasis of, 466; on its effects on the joints, 469; observations on the comparative advantages of the cooling and the heating treatment of, xxxviii. 283; general observations on, xxxix. 251; case of chronic, successfully treated by bandages, xl. 305.
RICHERAND, M. case of excision of a portion of the ribs and pleura, xl. 272.
RICKETS, observations on the origin and treatment of, xxxvii. 325.
RHINOCEROS, account of a fossil, xxxvii. 522.
RIBS, observations on fracture of the, xxxviii. 475; account of the removal of a portion of the, xl.
ROBERTS, Mr. case of cynanche laryngea, xxxv. 413.
ROBERTON, Dr. remarks on the dedication of his book to Dr. Baillie, and on the nature of its contents, xxxvi. 20.
ROBIQUET, M. account of his mode of producing prussic acid, xl. 449.
ROCHON, M. on a mode of making ship-lanterns, xxxv. 336.
RODMAN, Dr. account of a fœtus of the fifth month, preserved alive, xxxv. 424.
RONALDS, Dr. observations on the treatment of acute rheumatism, xxxv. 8; observations on measles, xxxvi. 10.
ROOTSEY, Mr. review of his General Dispensatory, xxxv. 141; remarks on the review of his work in the Repository, 275.
ROSE, Mr. remarks on his observations on the treatment of syphilis without mercury, xxxix. 224.
ROSTAN, M. observations on aneurism of the heart, xl. 443.
ROUX, M. observations on the treatment of paralysis, xxxvii. 157; on the operation for the cataract, xxxviii. 338; xxxix. 515.
ROWE, Mr. account of a case of obstinate costiveness, xxxix. 17; observations on the fevers of London, 39.
RUSH, Dr. Benjamin, accounts of the life and character of, xxxv. 366; xxxvii. 46, 48, 81, 338, 384, 448; xxxviii. 156.
RUST, Professor, account of a peculiar disease of the humerus, xxxviii. 355.

### S.

SABATIER, Professor, observations on curvature of the spine, xxxvii. 346.
SALISBURY, Mr. account of his botanical excursions, lectures, and observations, xxxv. 430, 516; xxxvi. 84, 176, 258, 348, 170, 349; xxxvii. 248, 340, 425, 539; xxxviii. 86, 349, 437.
SALIVATION, instance of its occurrence in a suckling infant, from the milk of the mother, xxxv. 512.
SALMON, Mr. account of some instruments for supporting the head, xxxvii. 266.
SALT, Mr. on constitutional diseases, communicated by inoculation, xxxvii. 517.
——, the mineral, observations on its medicinal qualities, and on the effects of the duties of it on the health of the people, xxxix. 289, 291, 292, 294, 368, 434.

SANCTIS, Dr. de, account of his description of a lusus naturæ, xxxix. 67.

SANDARS, Mr. observations respecting Miss M'Avoy, xxxix. 114.

SATTERLY, Dr. cases of diabetes, xxxvi. 115.

SAUNDERS, Dr. account of the life of, xxxviii. 388.

SAUSSURE, Professor, account of his illness and death, xxxvii. 70.

SAWS, description of some old chirurgical, xxxvii. 265.

SCARIFICATOR, description of a new one, xxxvii. 81.

SCARPA, Professor, on his improved operation for aneurism, xxxvii. 377; on the operation for the artificial pupil, 496.

SCHMIDT, Dr. notice of his work on the Pathology of the Spleen, xxxix. 239.

SCIATICA, on the effect of stramonium, in the cure of, xxxvii. 337; on the efficacy of the actual cautery, in the cure of, xxxix. 257.

SCOTLAND, remarks on the frequency of insanity in, xxxvii. 409.

SCOTT, Dr. on the use of the nitro-muriatic acid bath, in the cure of several diseases, xxxviii. 466.

———, Mr. James, observations on fever, xl. 276.

SCROFULA, remarks on its nature and origin, xxxv. 329; remarks on its frequency in deaf and dumb persons, xxxvii. 258; general observations on, 335; observations on the effects of an aqueous regimen, in, 394.

SCUDAMORE, Dr. review of his work on Gout and Rheumatism, xxxvi. 498.

SCURVY, observations on the treatment of sea, xxxv. 257; on the use of the acid of sugar, in the cure of, 338; on the nature of the hæmorrhage, in, 408.

SECRETION, account of some experiments to determine the influence of nerves, in, xxxv. 331; observations on that of fat, xxxvi. 304; various observations on the, 340; account of the different opinions respecting that of the milk, 432; cases of change of the colour of that of the dermoid organ, xl. 391, 392.

SENSATION, uneasy, considered as the cause of muscular motion, generally, xxxv. 261; observations on the connexion of morbid, with madness, 492; remarks on some opinions of Hartley, xxxix. 98.

SENSIBILITY, remarks on the nature of morbid, xxxv. 11; on the difference of that of the nervous system of animal and of organic life, 53; on its relation to muscular motion, 260; observations on its connexion with madness, 492; observations on its properties and origin, xxxvi. 128; observations on its state in mania, xl. 124.

SEROUS EFFUSION, observations on the origin of, xxxvii. 311.

SERPENTS, examination of some remedies employed for the bite of poisonous, in Ceylon, xxxix. 284.—*See* VIPER.

SERRES, M. history of a case of aphony, xxxvi. 164; observations on the origin of paralysis from solution of continuity of the brain, xl. 351.

SHATH, Dr. observations on the treatment of puerperal fever, xxxvi. 1; cases in midwifery, 272.

SHEEP, case of hydrocephalus in a, xxxvii. 431; on the rot, xxxix. 433.

SHEPHERD, Mr. observations on the mercurial treatment of the yellow-fever, xxxviii. 487.

SHOULDER, case of amputation at the joint of the, xxxvi. 108.—*See* AMPUTATION.

SKIN, account of a change of the colour of the, xxxvii. 152, 209; xl. 391; on contractions of the, from the cicatrices of ulcers, 510.—*See* CUTANEOUS DISEASES.

SLEEP, account of a woman who habitually slept for a very long period, xxxv. 509; remarks on the means of remedying the want of, xxxvi. 398.

SMALL-POX, observations relative to the laws respecting inoculation with, xxxv. 66, 155, 202; its great prevalence in London, in 1816, 79; remarks on its inflammatory nature, 365; observations on the ordinary progress of, in its distinct and confluent forms, 439; case of secondary, 477; miscellaneous observations, in the manner of axioms, on, xxxvi. 14; remarks on those observations, 112; cases of, after vaccination, xxxvii. 2; cases of natural, ibid.; remarks on those cases, 8; history of the recurrence of, in the same person, 27; report of the hospital for, in 1816, 254; remarkable case of, 255; occurrence of, after vaccination, 436; report of the hospital for, in 1817, xxxviii. 81; remarks on its occurrence after cow-pox, 107; report of the hospital for, xxxix. 81; remarks on the history of, 265; account of the epidemic appearance of it in Cupar, in Fife, 509; observations respecting its analogy to chicken-pox, xl. 457.

SMERDON, Mr. history of a case of diabetes, xxxvi. 26; on the distinction between healthy and diabetic urine, 31; further observations on diabetes, xxxvii. 104, 186.

SMITH, Dr. case of lithotomy, xxxix. 408.

———, Dr. of New-York, observations on the use of emetics, in spasmodic diseases, xl. 326.

SMITHSON, Mr. on the colouring properties of some vegetable substances, xxxix. 253.

SNAILS, account of their mode of breeding, xxxix. 400.

SNAKE, account of one found in a block of coal, xxxvii. 431.

SOCIETY, *of Sciences, at Copenhagen;* presentation of a waxen model of the proteus anguinus, a piece of aërolite, and a new fossil, by Mr. Wadd, xxxv. 69.

———, *Royal Geological, of Cornwall;* observations on the tests for arsenic, by Dr. Paris, xxxviii. 172.

———, *the Association of the Fellows and Licentiates of the Dublin College of Physicians,* vol. i. containing, the history of a case of ulceration and rupture of the stomach, by Dr. John Crampton, xl. 293; cases of tumors within the abdomen, by Dr. Stoker, 297; case of suppuration of the liver, by Dr. O'Brien, 298; dissections of two habitual drunkards, by Dr. Black, 299; case of gouty affection, by Dr. Black, 300; a case, shewing the influence of hepatic disease over the functions of the uterus, by Dr. Mills, 301; a case of hydrocephalus, by Dr. Brooke, ibid.; on the use of oxygen gas in angina pectoris, by Dr. Reid, 303; on the nature and treatment of tetanus, by Dr. Reid, ibid.; three cases of melæna, by Dr. Brooke, 304; on dropsy and apoplexy, by Dr. Stoker, 305; a case of chronic rheumatism cured by bandages, ibid.; a case of hydrocephalic fever, by Dr. John Crampton, 306; a case of abscess in the liver, by Mr. Geoghegan, ibid.; a case of complicated dropsy and dissection, by Dr. M'L[illegible]hlin, ibid; observations on the epidemic fevers of Dublin, by Dr. Edward Percival, ibid.; medical report of the fever-hospital, in Cork street, Dublin, by Dr. O'Brien, 398; medical report of the fever-hospital, in Cork-street, Dublin, by Dr. Grattan, 399; abstract of a registry kept for some years in the Lying-in Hospital of Dublin, by Dr. Clarke, 401.

SOCIETY, *Royal, of Edinburgh,* account of the sleeping woman of Dundonald, 509; account of the operation of the waters of the ocean and of the river Dee, in the harbour of Aberdeen, by Mr. Stephenson, xxxviii. 171; on the relation in the law of definite proportions, in chemical combinations, to the constitution of the acids, alkalies, and earths, and their compounds, xxxix. 430.

———, *National Institute of France,* report on a new translation of the works of Euclid, by M. Peyrard, xxxv. 245; account of the fall of some meteoric bodies, by M. Pistolet, 423; account of some of the transactions of, in 1816, xxxviii. 238; a memoir on contagion, 345; observations on the operation for the cataract, by M. Roux, xxxix. 515.

———, *Medical Society of Emulation, of Paris,* a memoir on the earth-eaters of Africa, xxxviii. 79.

———, *of Science, of Haerlem,* prize-question of, in 1816, xxxv. 70.

———, *Medical, of London,* anniversary meeting in 1816, oration by Dr. Clutterbuck, xxxv. 333; anniversary oration in 1817, by Mr. Stevenson, xxxvii. 390; account of the death of Dr. Rush, by Dr. Mease, xxxvii. 81; anniversary meeting in 1817, 330; transactions of, vol. i. part 2, containing a paper on epilepsy, by Dr. Adams, 126; observations on the treatment of croup, by Mr. Blegborough, 126; observations on croup, by Mr. Gaitskell, 128; account of three cases of extraordinary periodical sickness, two of which were cured by arsenic, by Dr. Adams, 129; case in which nearly an ounce of sulphuric acid had been swallowed, by Dr. Adams, 129; a case of lusus naturæ of the female organs of generation, by Mr. Gaitskell, 130; case of vermis lumbricus perforating the intestinal canal and abdomen, by Dr. Lettsom, 128; case of diseased action of the heart, effectually relieved by blood-letting, by Dr. Clutterbuck, 130; annual meeting of, in 1818, oration by Dr. Uwins, xxxix. 335.

———, *Royal, of London,* annual meeting and transactions of, in the year 1816, xxxv. 69; account of the medical and physiological part of the transactions of, in 1815, 119; some additional experiments and observations on the relation which subsists between the nervous and sanguiferous system, by Dr. A. P. Wilson Philip, 119; account of Dr. Clanney's safety-lamp for mines, 243; account of some experiments on the nervous influence in secretion, by Dr. Philip, 331; observations on the torpedo, by Mr. Todd, 333; account of the structure of the feet of some lacertæ, 333; observations on the specific actions of medicines, by Sir E. Home, 422; account of a case of aphony, cured by electricity, xxxvi. 664; observations on the formation of fat in tadpoles, by Sir E. Home, 165; on the application of galvanism to the cure of certain nervous diseases, by Dr. Wilson Philip, 165; account of some experiments on the physiology of plants, by Mr. Knight, 166; experiments on the eyes of fishes, by Dr. Brewster, 166; a further account of the mechanism by which insects can walk contrary to their gravity, by Sir Everard Home, 258; account of the mode of fumigating infected letters, by Dr. Gomes, ib.; observations on the means of remedying the must of corn, by Mr. Hatchett, xxxvii, 156; observations on the passage of the ovum into the uterus, by Sir Everard Home, 76; xxxix. 395; on an improved method of preparing the colchicum for medical use, xxxviii. 76; proceedings on the re-commencement of the sittings of the, in December 1818, xxxix. 257; observations on the composition of the blood, by Sir E. Home, 258; observations on the urinary organs, and secretion of some of the amphibia, by Dr. John Davy, 514; account of a malformation of the uterus, by Dr. Granville, 514; account of the production of sulphuretted azote in the abdomen, by Dr. Granville, xl. 165; account of a new principle, obtained from uric acid, by Dr. Prout, 166.

————, *Medico-chirurgical, of London*, transactions of the, vol. vi.; account and origin of the plague at Malta, by Dr. Calvert, xxxv. 319; observations on some affections of the larynx, which require the operation of bronchotomy, by Mr. Lawrence, xxxvi. 61; observations on the Mediterranean fever, by Dr. Denmark, 155; experiments on the formation of bone, by Mr. Howship, 157; transactions of, vol. vii. part 1, containing chemical analysis of the mineral waters of Spa, by Dr. Jones, 481; history of two cases of angina pectoris, 481; history of a very fatal affection of the pudendum of female children, by Mr. Kinder Wood, 482; cases and observations, illustrating the influence of the nervous system in regulating animal heat, by Mr. Henry Earle, 486; an account of the last illness and death of Professor de Saussure, by Dr. Odin, xxxvii. 70; history of a case of chorea, by Mr. Kinder Wood, 151; description of a monstrous fœtus, ib.; observations on a change of colour of the skin, by nitrate of silver, by Dr. Albers, 152; case of un-united fracture of the os humeri, successfully treated by the seton, by Mr. Stansfield, 152; case of a wound of the face, in which the carotid artery was tied, by Mr. Collier, 152; history of a tumor of the face and neck, in which the carotid artery was tied previously to its removal, 152; history of a case of aneurism of the femoral artery, cured by ligature of the external iliac, 154; observations on the treatment of varicose veins of the legs, by Mr. Brodie, 155; case of inflammation of the muscular structure of the heart, by Mr. Stanley, 323; sketch of the medical history of the first battalion of the first regiment of the foot-guards, during the winter of 1812 and 1813, by Mr. Bacot, 325; microscopic observations on the structure of bone, by Mr. Howship, 325; observations on rickets, by Mr. Stanley, 352; observations on tetanus, by Dr. Dickson, 632; account of a case of curious imperfection of vision, by Dr. Nicholl, 331; observations on consumption, by Dr. Philip, 331; observations on the venereal disease in the fœtus, by Mr. Hay, 326; on the medicinal properties of stramonium, by Dr. Marcet, 337; observations on the artificial pupil, by Professor Scarpa and M. Mannori, 496; case of a wound of the peroneal artery, by Mr. Guthrie, 500; case of a gun-shot wound, and fracture of the tibia, by Mr. Boggie, ib.; an account of a new method of operating for the cure of external aneurism, by Mr. Crampton, ib.; further observations on contraction succeeding to ulceration of the skin, by Mr. Earle, 510; case of hernia of the dura mater, connected with hydrocephalus, by Mr. Earle, 510; description of an extra-uterine fœtus, contained in the fallopian tube, by Mr. Langstaff, 510; on the treatment of sinuous ulcers, by Dr. Dewar, 513; of Mr. Salt's essay on the mode by which constitutional disease is produced from the inoculation of morbid poisons, 517; of Dr. Cruveilhier's essay on pathological anatomy, 519; of Dr. Gardanne's advice to women, 520; transactions of vol. viii. part 1, report of the state of the wounded on-board his majesty's ship Leander, in the action before Algiers, by Dr. Quarrier, xxxviii. 411; cases of hernia cerebri, with observations, by Mr. Stanley, xxxix. 71; history of a case of rupture of the brain and its membranes, by Dr. Baron, xxxviii. 464; history of a case of ill-conditioned ulcer of the tongue, successfully treated by arsenic, by Mr. Lane, xxxix. 76; history of a case of lithotomy, with a few remarks on the best mode of making the incision in the lateral operation, by Mr. Samuel Cooper, 76; case of fatal hæmorrhage from the extraction of a tooth, by Mr. Blagden, 76; account of some remarkable symptoms which were connected with a painful affection of the extremity of the left thumb, by Mr. John Pearson, 77; observations on the morbid structure of bones, and an attempt at an arrangement of their diseases, 145; an enquiry into the origin and nature of the yellow-fever, as it has lately prevailed in the West Indies, with official documents relating to this subject, 149; case of rupture of the stomach, and escape of its contents into the cavity of the abdomen, by Dr. John Crampton, 154; cases of fungus hæmatodes, by Mr. Langstaff and Mr. Lawrence, 155; on the pellagra, by Dr. Holland, 210; observations on the treatment of syphilis, with an account of several cases of that disease, in which a cure was effected, without the use of mercury, by Mr. Rose, 223; observations on the treatment of the venereal disease, without mercury, by Mr. Guthrie, 223; transactions of vol. viii. part 2, observations on the nature of some proximate principles of the urine, with a few remarks upon the means of preventing those diseases connected with a morbid state of that fluid, by Dr. Prout, 322; account of three cases of calculi, removed from the urethra without the use of cutting instruments, by Mr. Astley Cooper, 417; some cases of disease of the heart, by Mr. James, 418; further observations on the ligature of arteries, by Mr. Lawrence, 427; a case of extra uterine fœtus, by Mr. Langstaff, 429.

SOCIETY, *Medical Benevolent, of London*, account of, xxxv. 246, 509; xl. 74.

——, *Imperial, of Naturalists, at Moscow*, account of its institution, xl. 254.

——, *Philosophical, of London*, anniversary meeting, in 1817, xxxviii. 78.

——, *Linnæan, of London*, account of the ancient inhabitants of Guadaloupe, xxxv. 244; description of the *saracenia flava* and adunca, 244; account of a pig being found alive in the interior of a chalk-hill, 511; observations on the sea long-worm of Borlase,

5

*gordius marinus* of Montagu,' by the Rev. Mr. Davies, xxxvi. 207; a tabular view of the external characters of four classes of animals which Linnæus arranges under *insecta*, &c. by Dr. Leach, 209; description of a fossil alcyonium, from chalk strata near Lewes, by Mr. Mantell, 212.

SOCIETY, *Truss, of London*, report of, in 1815; xxxv. 246; report of, in 1817; xxxviii. 336; xxxix. 170.

——, *Wernerian*, account of some fossil remains, found at Kilmarnock, xxxix. 431; account of an ourang-outang, by Dr. Traile, 431.

——, *Physico-Medical Society, of New-York*, vol. i. observations on certain causes which influence the decarbonizing function of the lungs, by Dr. Pierson, xl. 331; observations on cynanche laryngea, by Dr. Bliss, 335; observations on the efficiency of emetics in spasmodic diseases, by Dr. Smith, 336; a case of sudden death, from a rupture of the left ventricle of the heart, by Dr. Mott, 425; case of phlegmasia dolens in a male, by Dr. Purdy, 426; case of artificial joint, cured by friction, by Dr. Meeker, 427; an account of a family predisposition to hæmorrhage, by Drs. W. and J. Buel, 429; reflections on the pulsation in epigastrio, by Dr. Mott, 527; case of brachial aneurism, cured by tying the subclavian artery above the clavicle, by Dr. Post, 531.

——, *Medical, of Paris*, account of a case of general transposition of the thoracic and abdominal viscera, by M. Beclard, xxxvii. 157; a memoir on the treatment of coxalgia and rachialgia, by M. Le Baron Larrey, 158.

——, *of Sciences, at Rouen*, prize question of, for 1816, xxxv. 70.

SODA, observations on the effects of it on the animal fibre, xxxvii. 68; the muriate of, recommended for the preservation of anatomical preparations, 180, 426; on the use of the carbonate of, as an application to ulcers, xl. 207.

SODAR, Mr. case of inguinal aneurism, xxxvii. 509.

SOLAR SPECTRUM, on the magnetizing power of the violet rays of the, xxxix. 524.

SOMERVILLE, Dr. description of the female Hottentot, xxxvi. 466; on the virtues of the pyrola umbellata, 471.

SOUND, for the bladder, description of an improved one, xxxv. 81.

SOUTHEY, Dr. observations on elephantiasis, xxxv. 213.

SPASM, observations on its influence in the production of disease, xl. 247.

SPECIFIC MEDICINES, observations on their effects on the animal economy, xxxvi. 506.

SPEECH, observations on some defects in the powers of, xxxxi. 226; xxxix. 375.

SPEN, Dr. case of disease of the heart, xxxvi, 51.

SPENCER, Mr. observations on some improved surgical instruments, xxxvii. 265.

SPIDER, account of a worm found in the belly of a, xxxix. 478.

SPINA BIFIDA, description of a case of, xxxix. 183.

SPINE, observations on inflammation of the membranes of the, xxxv. 339; cases of disease of the bones of the, xxxvi. 279; observations on caries of the, xxxvii. 158; on the treatment of curvature of the, 346; on the origin of epilepsy from disease of the, 261; observation on diseases of the, xxxviii. 245; xl. 450.

SPINAL MARROW, experiments and observations on its influence on the circulation of the blood, xxxv. 119; remarks on the influence of the, on the action of the heart, 318; account of some experiments on the destruction of the, to determine its influence on the functions of the intestinal canal, 332; on inflammation of the membranes of the, in children, 339; observations on the connexion of disease of, with palsy, xxxix. 5; observations respecting the origin of tetanus from disease of the spinal marrow, xl. 259, 303; account of the opinions of several physiologists respecting the cause of its destruction in the fœtus, 432; observations on the cause of the cavity formed in it, 432; observations on the connexion of disease of the, with fever, 321; cases of tetanus, originating from disease of the, 259, 303.

SPLEEN, observations on a morbid state of, in a case of puerperal fever, xxxvii. 117; observations on the pathology, xxxix. 237.

SPURZHEIM, Dr. observations on the doctrine of, xxxvi. 251; xxxvi. 427; xxxvii. 160, 220; xxxviii. 29; xxxix. 4.

SQUIRE, Dr. biographical account of, xxxvi. 257, 347.

STANFIELD, Mr. case of fracture of the os humeri, xxxvii. 152.

STAFFORD, Mr. account of the plague at Malta, xxxv. 152.

STANLEY, Mr. case of inflammation of the heart, xxxvii. 333; on the state of the bones in rickets, 325; cases of hernia cerebri, xxxix 71.

STARR, Mr. observations on the propriety of bleeding in some species of injury, xxxvii. 97.

STEINBUCH, Dr. account of a new remedy for the tooth-ache, xxxvii. 262.

STEVENSON, Mr. on the connexion of diseases of the eye with a morbid state of the intestines, xxxvi. 442; account of a new instrument for the eye and ear, xxxvii. 81; observations on the operation for the cataract, xxxix. 177.

STEWART, Dr. Duncan, review of his treatise on uterine hemorrhage, xxxvi. 489.

———, Mr. Jas. account of a case of disease of the heart, xxxvii. 416.

STIMSON, Dr. history of an extraordinary case of diarrhœa, xxxviii. 193.

STOKER, Dr. histories of some cases of tumors within the abdomen, xl. 315.

STOMACH, case of inflammation of, subsequent to puerperal fever and phlegmasia dolens, xxxv. 14; remarks on the connexion of disorders of, with hydrocephalus, 90; on the influence of disorder of, on the brain, 302; account of some experiments on the influence of the nerves on the exercise of its functions, 331; observations on the peculiar form of inflammation of the, produced by arsenic, 338; case of hernia of the, through an opening in the diaphragm, xxxvi. 232; observations on its connexion with gout and rheumatism, 500; remarks on craving sensations of the, xxxvii. 209; account of a polypus of the, 433; observations on wounds of the, xxxviii. 370; observations on a disease of the stomach and intestines sometimes mistaken for syphilis, xxxix. 412; case of rupture of the, 154; observations on the glands of the, 402; observations on the treatment of spasms of the, 453; observations on the state of the, in the act of vomiting, 517; observations on its state in fever, 50, 51, 60, 61; case of ulceration and rupture of the, xl. 295; observations on rupture of the, from the act of vomiting, 532; observations on its functions in digestion, 533; observations on the elective action of the stomach in digestion, 531, 534, 535, 536, 537.

STRABISMUS, observations on its causes in children, xxxv. 241. — *See* EYES.

STRAMONIUM, account of its utility, in a case of mania, xxxv. 338; observations on its medicinal qualities, xxxvii. 28, 237.

STRICTURES, *of the œsophagus*, cases of, xxxv. 346.
———, *of the urethra*, on the use of tobacco glysters, in the treatment of, 408; various observations on the treatment of, xl. 140.
SUCK, Dr. observations on a case of retroversion of the pupil of the eye, xxxvi. 346.
SUGAR, chemical observations on, xxxix. 293.
SULPHUR, observations on the medicinal qualities of the liver of, xxxvi. 373; on the use of fumigation with, in various diseases, xxxvii. 85.
SULPHURET OF POTASH, observations on its efficacy in croup, xxxvi. 373.
SUN, observations on the benefit resulting from the influence of its beams on the body, in some diseases, xxxv. 157, 159.
SURGERY, historical observations on, xxxv. 249; extracts from very early English works on, 311, 381; account of Assalini's improved instruments for operations in, 396; observations respecting the state of, amongst the ancient Egyptians, xxxvi. 448; account of the first operation for the stone, 519; account of a general treatise on, xxxvii. 351; miscellaneous observations relative to, xxxviii. 49, 62; statistical observations respecting operations in, 420; observations on military, xl. 154.
SUTLIFFE, Mr. account of a case of puerperal peritonitis, xxxix. 513.
SUTTON, Dr. observations on pulmonary consumption, xxxv. 182; xxxvii. 95, 350, 456.
SWALLOWS, observations respecting the hybernation of, xxxviii. 519.
SWEDIAUR, Dr. his account of the venereal disease of Canada, xxxix. 209.
SYDENHAM, his remarks on the use of bleeding, in gout, xxxv. 414; on bleeding from the vessels under the tongue, in angina, 416; extract from his observations on measles, xxxv. 11; remarks on his character, xxxvii. 48; remarks on the constitution of the atmosphere, xxxix. 262; on the use of opium, 448.
SYER, Mr. observations on fever, xxxix. 362.
SYM, Mr. observations on the temperature of water, xxxvii. 525; observations on fever, xl. 321.
SYMPATHY, observations relative to sympathetic affection of the heart with the brain, 319; various remarks on, 458; observations on its nature and causes, xxxvi. 124; observations on that existing between the eye and the intestines, 346; general observations on its agency in the production of disease, xl. 242, 405; observations respecting that existing between the throat and the genitals, 507; some observations showing the agency of, in producing death, in cases of severe injury of some part of the nervous system, 440.

### T.

TADPOLES, observations on the growth of, xxxvi. 165.
TABES MESENTERICA, observations on, with cases connected with dropsy and hydrocephalus, xxxix. 93, 95.
TALLIACOTIAN ART, observations on the revival of, xxxv. 293.
TAR, observations on the efficacy of the vapour of, in the cure of some affections of the lungs, xxxix. 511; xl. 82.
TAUNTON, Mr. account of the juridical process against, on the subject of small-pox inoculation, xxxv. 66, 155; observations on the use of opium, in tetanus, xxxvii. 371; report of the London Truss Society, xxxix. 170.—*See the article* SOCIETIES.
TEA, observations on the medicinal qualities of, xxxix. 495.
TEETH, account of a new instrument for the extraction of, xxxv. 433; on the preservation of the, xxxvi. 255; account of a peculiarity for, in the structure of the, xxxvii. 89, 441; on the extraction of the, 443; case of fatal hæmorrhage from the extraction of a tooth, xxxix. 76.
TEISSIER, M. observations on the duration of utero-gestation, and of incubation of various animals, xxxix. 6.
TEMPERATURE, observations on the effects of, produced by the rays of the sun, in the cure of several diseases, 156; observations on the methods of increasing the external, for the cure of some diseases, 169; on the utility of low degrees of, in various pulmonary diseases, 182; observations on its influence on consumption, xxxviii. 97; observations respecting the influence of climate on that of animals, xl. 451.
TESTICLES, review of a treatise on the diseases of the, xxxv. 218; case of atrophy of the, xxxvi. 174; case of inflammation of the, 178; case of wound of a testicle, followed by the formation of a bony substance, xxxix. 341.
TETANUS, case of supposed, in which a glyster of oil of turpentine seemed to effect a cure, xxxv. 408; case of, treated by copious bleeding, and tobacco glyster, 410; termination fatal, ibid.; account of its origin and treatment, in the children of the Caribbees, 475; history of two cases of, xxxviii. 19; review of a treatise on, 317; remarks on idiopathic, 318; on the distinction between rabies and, 321; cases of, 322, 329; on the benefits of large doses of opium, in, 371; observations on that of infants, especially on its connexion with disease of the umbilical cord, xxxix. 497; observations respecting its origin from disease of the spinal marrow, xl. 259, 303; case of locked jaw accompanying hysteria, 15; observations respecting its origin from gun-shot wounds, 220; on the division between spontaneous and taumatic, 220; cases originating from diseases of the spinal marrow, 259, 303; case of locked-jaw from cancer, 386; case of trismus accompanying hysteria, xl. 15.

THACKER, Dr. observations on puerperal fever, xxxv. 14.

THERAPEUTICS, review of a general treatise on, xxxvii 51, 127.

THERMOMETER, description of a new one, xl. 92.

THILENIUS, Dr. account of his medico-chirurgical work, xxxvi. 510; observations on the tooth-ache, xxxvii. 262.

THOMAS, Mr. H. L. account of a case of obstruction of the intestines, xxxvi. 67.

THOMPSON, Dr. on the cure of syphilis without mercury, xxxix. 240.

THORAX, case of transposition of the viscera, xxxvii. 157, 346; on gun-shot wounds of the, 97; on wounds of the, 29.

THROAT, case of inflammatory affection of the, xxxvi. 47.

TIC DOULOUREUX, observations on the origin of, xxxvii. 137; on the efficacy of stramonium, in the cure of, 337; on the use of belladonna, in the cure of, xxxix. 170; observations on, 283; on the efficacy of tar, in the cure of, 519; of the use of calomel and opium, in the cure of, xl. 476 —*See* Neuralgia.

TINEA CAPITIS, observations on the efficacy of turpentine, in the cure of xxxvii. 101.

TOAD, account of one found alive in a stratum of rock, xxxviii. 35.

TOBACCO, account of the deleterious effects of, xxxv. 151; observations on its use, in retention of urine, 408.

TODD, Mr. account of the torpedo, xxxv. 333.

———, Mr. of Dublin, account of an enlargement of the biliary ducts, xxxix. 499; observations on hernia, xl. 151.

TONGUE, account of abscess of the, xxxv. 250; case of paralysis of the, cured by galvanism, xxxvi. 164.

TONICS, observations on their effects on the animal fibre, xxxvii. 63.

TORPEDO, some observations on the, xxxv. 333.

TOUCH, account of some phenomena respecting the power of, xxxviii. 91, 366; xxxix.

TOURNIQUET, some observations on the use of the, xxxviii. 63.

TRACHEA, observations on inflammation of the, xxxvi. 307, 405; xxxviii. 51; cases of foreign bodies lodged in the, xxxix. 100, 102.—*See* Croup, *and* Larynx.

TRAIL, Dr. description of the oran outang, xxxix. 431.

TRAVERS, Mr. observations on the ligature of arteries, xxxvi. 235; review of his Surgical Essays, xl. 502.

— ———, Dr. case of ossification of the larynx, xxxvii 155.

TRIBOLET, Dr. on the use of henbane in inflammation, xxxvii. 163.

TRISMUS NASCENTIUM, observations relative to its origin, xxxix 497.

TROPICS, observations on the endemics of the, xxxvii. 233.

TROTTER, Dr. observations on sea scurvy, xxxv. 257, 259; observations on diabetes, xxxix. 366.

TUMORS, account of a large encysted, connected with the kidneys, xxxv. 1; cases of, in the substance of the brain, 301, 303; case of, in the abdomen of a girl two years and a half old, containing part of a fœtus, 411; case of large, situated on the face and neck, removed after ligature of the carotid artery, xxxvii. 152; case of polypus in the heart, 101; case of polypus in the stomach, 433; case of large fungous tumor of the eye, xxxviii. 179; case of, apparently cancerous, adhering to the liver, 344; case of osteo-steatomatous tumor of the os humeri, 355; observations on the nature of fungus hæmatodes, 431; account of a large steatomatous, removed from the head, 517; cases of fungus hæmatodes, xxxix 155; case of large steatomatous, seated in the neck, xl. 62; histories of several, situate in the abdomen, 297; account of some found in the livers of habitual drunkards, 299; case of cartilaginous, of the os humeri, 364; case of large steatomatous, situate at the angle of the jaw, 62.

TURPENTINE, instance of the efficacy of the oil of, in a case of locked-jaw, xxxv. 408; instance of its efficacy, in a case of severe abdominal obstruction, xxxvi.359; on its use, in the cure of tinea capitis, xxxvii. 102; observations on its efficacy in the cure of puerperal peritonitis, xxxviii. 447; observations on its efficacy, in the destruction of tæniæ, xl. 546; in melæna, 304.

TURTLE, observations on the blood of the, xxxvii. 388.

TYMPANITIS, observations on the efficacy of alum, in the relief of, xxxv. 73.

TYMPANUM, observations on the puncture of the membrane of the, xxxv. 253; xxxix. 16.

U.

ULCERS, observations on the treatment of, especially by pressure, xxxv. 418; observations on ulceration of the cartilages of the joints, xxxvi. 229; case of ulceration of the pharynx, 402; case of ulceration of the larynx, 404; case of ulceration of the larynx and trachea, 405; history of some fatal cases of, affecting the pudendum of female children, 482; observations on the contraction of the skin on the cicatrization of, xxxviii. 510; observations on the treatment of fistulous, 513; case of ulceration of the stomach, terminating in rupture of its coats, xxxix. 154; observations on the ulceration of the umbilical cord of infants, 497; observations on that arising from hospital gangrene, 216, 219, 226, 239.

UPAS ANTIAR, observations on the effects of the, on the animal economy, xxxv. 330.

URETHRA, observations on the diseases of the, xl. 139.

URINE, observations on diabetic, xxxvi. 31; on the qualities of, in gout, 503; observations on that in diabetes, 107, 187; observations on it, in various states, xxxix. 322, 455; experiments and observations on the influence of different species of food on the qualities of the, xl. 340; observations on the composition of lithic acid, 338; observations on cystic oxide, 339.

URTICATION, observations on its efficacy, in the relief of some diseases, xxxv. 73.

UTERUS, observations on extirpation of the, xxxv. 328; further observations on extirpation of, and excision of parts of the, 426; case of rupture of the, in the seventh month of pregnancy, 449; case of inflammation of the, occurring subsequently to artificial delivery, 462; observations on cancer of the, xxxvi. 250; observations on injection of the, in some diseases, 272; observations on cancer of the uterus, and on excision of the, 293, 294, 295; on the efficacy of bleeding and opium, in violent contraction of the, 331; observations on the specific action of ergot, on the, 379; observations on the treatment of hæmorrhage from the, especially by opium, 489; observations on the use of æther in the treatment of, 514; observations on its state, in puerperal fever, xxxviii. 117; case of section of the, through the vagina, 365; observations on hæmorrhage from the, 532; account of a severe disease, apparently from hydatids of the uterus and ovary, xxxviii. 58; observations on the passage of the ovum into the, 76; observations on hæmorrhage from the uterus, after delivery, 462; case of malformation of the, xxxix. 514; case of inflammation and gangrene of the, 344; observations respecting the formation of the deciduary membrane in the, in cases of extra-uterine fœtation, xl. 344; case of ovarian dropsy, 306; case of porlapsus of the, for which extirpation of that organ was performed, 325.

UWINS, Dr. observations on the medical effects of digitalis, xxxix. 91; observations on hydrocephalus, xxxvi. 100; extract from his oration before the London Medical Society, xxxix. 335.

UVA URSI, observations on its botanical character and medicinal qualities, xl. 488.

V.

VACCINATION, observations on the state and progress of, xxxv. 95, 98, 161, 477; xxxvi. 9, 17, 167, 169, 174; xxxvii. 2, 8, 158, 254, 374, 531; xxxix. 20, 160, 265.

VAGINA, case of incision of the uterus through the, for the delivery of a fœtus, xxxvii. 365; observations on hæmorrhage from the, 532; case of retroversion of the, xxxviii. 342; on serous discharge from the, xxxix. 428.

VALENTIN, M. observations on the nature and origin of yellow-fever, xxxvii. 43.

VARENNES, Mr. account of a woman who fasted eleven years, xxxviii. 341.

VAUQUELIN, M. analysis of some aërolites, xxxv. 423.

VENEREAL DISEASES, observations respecting the transmission of syphilis from the mother to her infant, xxxv. 512; some observations on, and especially on those termed cachexia syphiloidea, xxxvi. 333; experiments and observations on the nature and effects of the matter of syphilitic buboes, xxxvii. 37; observations on the use of the mineral acids, in the treatment of, xxxviii. 429; various observations on the treatment of, 478; observations on the cure of syphilis without mercury, and on some affections of the intestinal canal, mistaken for that disease, xxxix. 412; observations on the use of gold, in the treatment of, 515; remarks on those resembling syphilis, xxxix. 206; observations on the efficacy of opium, in the cure of, 207; observations on the treatment of, in Portugal, 228; observations on the treatment of syphilis, without mercury, 229, 236, 290, 412; observations respecting the analogy of the disease prevalent in the latter part of the 15th century, with sibbens, xl. 596; observations on syphilitic inflammation of the eyes, 504; some observations on the antiquity of its origin, 193; observations respecting the cure of syphilis without mercury, 5, 48, 170, 320, 340, 374, 470.

VETCH, Dr. remarks on Sir William Adams's treatment of ophthalmia, xxxix. 209.

VEINS, observations on varicose, and on a new method of treating those of the legs, xxxvii. 155; observations on distension of the, xxxviii. 374; remarks on the division of the saphena, xxxix. 516; observations on the effects of wounds and ligatures on them, xl. 521; on the mode in which union of wounds of them takes place, 523; on the causes and termination of inflammation of the, 524, 525.

VIPER, experiments with the venom of the, xxxvi. 522; xxxvii. 434; account of the effects of the bite of the, xxxviii. 517.

VIREY, Mr. observations on the ergot rye, xxxvii. 162; remarks on worms found in the human body, xxxviii. 432; on the medical measures of brute animals, xl. 486.

VISION, observations on nyctalopia, xxxvi. 362; case of curious imperfection in, xxxvii. 331; case of acquisition of, in a boy born with cataracts, xxxviii. 518; observations on some phenomena of, xxxix. 354; various remarks on, especially on the power of distinct, at different distances, xl. 177, 178, 179. —*See* EYES.

VOICE, case of loss of, removed by electricity, xxxvi. 169.—*See* SPEECH.

VOMITING, observations on the nature of the act of, xxxix. 517.

### W.

WADD, Mr. William, observations on hernia congenita, xxxv. 109; review of his work on Diseases of the Bladder and Testicles, 217; observations on the formation of fat, xxxvi. 303; remarks on corpulency, xxxvii. 245; review of his Cases in Surgery, xxxix. 304.

WALES, Mr. observations on the necessity of the profession of an accoucheur, xl. 17.

WALKER, Mr. Richard, observations on vaccination, xxxv. 93; on mania, 268; on the character and progress of the natural small-pox, 439; on the treatment of the small-pox, 445; observations on small-pox, xxxvi. 14; on the doses of medicine, 104; remarks on his observations on small-pox, 112; reply, 285; rejoinder, 375; answer, 458; observations on schirrus and cancer, 459; on the minim measure, 516; remarks on some of his papers, xxxvii. 103; reply, 193; rejoinder, 288, 423; his pharmaceutical table, 192; observations on the utility of linseed cataplasms, xxxix. 162; recipe for an adhesive powder, ibid.

WARD, Mr. H. remarks on the dropping of fluids, xxxvi. 426; on the medical qualities of the humulus lupulus, 456; remarks on the minim measure, xxxvii. 103, 112, 251; on the medicinal qualities of stramonium, 287; observations on the efficacy of nitric acid in diseases, xxxviii. 429; on the medical properties of the nitro-muriatic acid, xxxix. 339.

WARE, Mr. history of a case of polydypsia, xxxv. 199; observations on muscæ volitantes, xxxix. 103.

WARREN, Dr. observations on some diseases of the eyes, xxxvi. 382.

WATER, account of the composition of the Moira saline water, xxxvi. 428; of that of Cartmel, xxxvii. 267; on the means of rendering sea-water potable, 346; remarks on the influence of different species of, on the animal economy, 397; on the advantages of the use of distilled water, 406; on the density and temperature of, 525; some remarks on the influence of the rise of the sea on rivers, xxxviii. 171; account of the mineral waters of Caldas da Rainha, xl. 11.

WATERHOUSE, Dr. extracts from his Letters to Dr. Lettsom, xxxvii. 375.

WATSON, Dr. (Bishop of Llandaff,) memoirs of, xxxvii. 379.

WATT, Dr. observations on the hooping-cough, xxxix. 383.

WAYTE, Mr. further observations on the practice of midwifery, xxxv. 359.

WEST INDIES, observations on the endemic fever of the, xxxvii. 413.

WHATELY, Mr. review of his Treatise on Ulcers of the Legs, &c. xxxv. 418.

WHEELWRIGHT, Mr. history of a case of hernia, xxxvi. 232.

WHITFORD, Mr. description of a new tooth instrument, xxxv. 433.

WHITLEY, Mr. case of bronchotomy, xxxix. 100.

WHITLOW, observations on the treatment of it by pressure, xxxix. 185, 279.

WHITRIDGE, Dr. case of inguinal aneurism, xxxv. 368; case of amputation at the shoulder-joint, xxxvi. 108.

WILKINSON, Dr. account of lizards found in a chalk rock, xxxvii. 162; observations on the rise of fluids in capillary tubes, 426.

WILL, the, observations on its influence on muscular motion, xxxv. 262, 264; some observations on its influence on the organs of sense, the heart, &c. xxxvi. 92; observations on the power of the, on the body, with some curious illustrations, 393; instances of its power over the respiration and action of the heart, so as to induce apparent death, 394; observations on disordered impulses of the, in mania, xl. 122.

WILLAN, Dr. cases of phthiriasis, xxxv. 469; observations on measles, xxxvi. 12; observations on scarlet fever, xxxix. 499.

WILLIAMS, Dr. review of his Observations on Dr. C. Wilson's Specific for Gout, xl. 146.

WILSON, Mr. observations on the eggs of the frog, xxxix. 432.

WILSON, Dr. Andrew, review of his Letters on Morbid Sympathies, xl. 242, 404.

WIND OF A SHOT, observations on the imputed effects of the, xxxviii. 265, 373, 414.

WOOD, Mr. Kinder, account of a peculiar affection of the pudendum, xxxvi. 482; case of chorea, xxxvii. 151.

———, observations on the benefit derived from cold applied to the head, in typhous fever, xxxviii. 278; on the treatment of whitlow, xxxix. 273; experiments and observations on urine, 455.

WOODHAM, Mr. remarks on Mr. Walker's Observations on Small Pox, xxxvi. 112; reply to, 284; his rejoinder, 375; review of his remarks on Mr. Lawrence's lectures, 425; reply to Mr. Walker, xxxvii. 26; answer to, 193; his rejoinder, 288; his final answer, 425.

WOOLCOMBER, Dr. history of a case of cancer, xxxvii. 154.

WORMS, instance of their existence in the sinuses of the bones of the face, xxxv. 252; observations on the generation of the guinea-worm, 335; description of the sea long-worm, xxxvi. 207; account of one, found in the belly of a spider, xxxix. 478.

WOUNDS, observations on the hospital gangrene, consequent on, xxxv. 20; observations on the treatment of, especially by pressure, 418; observations respecting the reproduction of parts after, xxxvi. 19; observations respecting the adhesion of divided parts, 169; observations on the treatment of those arising from burns, 243; two cases of wounds of arteries in the operation of venesection, terminating without the formation of aneurism, 332; observations respecting adhesion of, by the first intention, 420; observations on the adhesion of, xxxvii. 515; cases of, affecting the thorax, 27; case of, in which the vertebræ were concerned, 98; a severe one of the face, for which the carotid artery was tied, 152; case of, affecting the shoulder-joint, 155; remarks on those of the cornea, 257; case of one, of the eye-brow, 260; case of, affecting the peroneal artery, 500; observations on the healing of, by adhesive inflammation, xxxviii. 11, 250, 251; case of wounded bladder, terminating favourably, 346; observations on those of the stomach, with cases, 370; account of several cases of, occurring during a naval combat, 411; observations on those affecting veins, xl. 521, 523.

WOUNDS, *gun-shot*, observations on the hospital gangrene, consequent on, xxxv. 20; case, in which a ball was found in the heart of a buck, xxxvi. 426; several cases of, especially affecting the thorax, xxxvii. 29; case of, of the shoulder-joint, 155; case of, injuring the tibia, 500; remarks on Dr. Alderson's claims to the modern improvement in the treatment of gun-shot wounds of the chest, xxxviii. 38; remarks on the period for amputation after the reception of, 63; case of, affecting the heart, xxxix. 404; case of extraordinary, of the head, 102; general remarks on, xl. 157; observations on the treatment of, 158, 160, 161, 162, 163, 167; on the proper period for amputation, in cases affecting the extremities, 159, 221; observations on the introduction of foreign bodies into, 161; on injuries of the bones in, 162, 163, 164; on contused wounds, from, 162; on injuries of the joints, from, 164; on injuries of the blood-vessels, from, 214, 215; on injuries of the nerves, from, 215; on tetanus, from, 220; on mortification, from, ibid.; on the hospital gangrene, consequent on, 216, 217, 218, 219, 226, 227, 228, 229, 230, 231, 232; historical observations respecting hospital gangrene from, 239; observations respecting tetanus as a consequence of, 220.

WRIGHT, Mr. notice of his work on Diseases of the Ear, xxxix. 139.

Y.

YEAST, remarks on its composition, xxxix. 303; observations on its use, in typhus, xl. 22.

YEATMAN, Mr. case of amputation at the instep, xxxvii. 353; remarks on the expediency of some legislative enactment, to insure competent medical aid to the sick poor, xxxviii. 364; xxxix. 331.

YEATS, Dr. remarks on hydrocephalus, xxxv. 88; reply to Dr. Monro's observations on the discovery of the action of the oblique muscles, attributed to Dr. Mayow, xxxvi. 251; remarks on hydrocephalus, xxxvii. 93; xxxviii. 26.

YOUNG, Mr. Samuel, remarks on his method of treating cancer, xxxvi. 460, 464; remarks on Mr. Cooper's operation for ligature of the aorta, xxxix. 180.

Z.

ZUGENBUHLER, Dr. observations on the active dilatation of the ventricles of the heart, xxxviii. 257.

FINIS.

Printed by J. and C. Adlard, 23, Bartholomew Close.

